# Single cell profiling at the maternal–fetal interface reveals a deficiency of PD-L1^+^ non-immune cells in human spontaneous preterm labor

**DOI:** 10.1038/s41598-023-35051-5

**Published:** 2023-05-16

**Authors:** Xiao Liu, Ivy Aneas, Noboru Sakabe, Rebecca L. Anderson, Christine Billstrand, Cristina Paz, Harjot Kaur, Brian Furner, Seong Choi, Adriana Y. Prichina, Elizabeth Ann L. Enninga, Haidong Dong, Amy Murtha, Gregory E. Crawford, John A. Kessler, William Grobman, Marcelo A. Nobrega, Sarosh Rana, Carole Ober

**Affiliations:** 1 Department of Human Genetics, University of Chicago, Chicago, IL USA; 2 Center for Research Informatics, University of Chicago, Chicago, IL USA; 3 Department of Obstetrics and Gynecology, Mayo Clinic, Rochester, MN USA; 4 Department of Immunology, Mayo Clinic, Rochester, MN USA; 5 Department of Obstetrics and Gynecology, Duke University Health Systems, Durham, NC USA; 6 Department of Pediatrics and Center for Genomics and Computational Biology, Duke University, Durham, NC USA; 7 Department of Neurology and Institute for Stem Cell Medicine, Feinberg School of Medicine, Northwestern University, Chicago, IL USA; 8 Department of Obstetrics and Gynecology, Feinberg School of Medicine, Northwestern University, Chicago, IL USA; 9 Department of Obstetrics and Gynecology, University of Chicago, Chicago, IL USA; 10 Present Address: Rutgers RWJ Medical School, New Brunswick, NJ USA

**Keywords:** Immunology, Medical research, Reproductive disorders

## Abstract

The mechanisms that underlie the timing of labor in humans are largely unknown. In most pregnancies, labor is initiated at term (≥ 37 weeks gestation), but in a signifiicant number of women spontaneous labor occurs preterm and is associated with increased perinatal mortality and morbidity. The objective of this study was to characterize the cells at the maternal–fetal interface (MFI) in term and preterm pregnancies in both the laboring and non-laboring state in Black women, who have among the highest preterm birth rates in the U.S. Using mass cytometry to obtain high-dimensional single-cell resolution, we identified 31 cell populations at the MFI, including 25 immune cell types and six non-immune cell types. Among the immune cells, maternal PD1^+^ CD8 T cell subsets were less abundant in term laboring compared to term non-laboring women. Among the non-immune cells, PD-L1^+^ maternal (stromal) and fetal (extravillous trophoblast) cells were less abundant in preterm laboring compared to term laboring women. Consistent with these observations, the expression of *CD274*, the gene encoding PD-L1, was significantly depressed and less responsive to fetal signaling molecules in cultured mesenchymal stromal cells from the decidua of preterm compared to term women. Overall, these results suggest that the PD1/PD-L1 pathway at the MFI may perturb the delicate balance between immune tolerance and rejection and contribute to the onset of spontaneous preterm labor.

## Introduction

The evolution of pregnancy in eutherian mammals required significant adaptations to provide for the survival of a genetically foreign fetus, including mechanisms allowing for maternal–fetal communication and bi-directional immune tolerance. The lack of rejection of the allogenic fetus has been referred to as the paradox of pregnancy^1^, which has remained unresolved for more than 60 years after it was first proposed^2,3^.

Human pregnancy differs from most other mammals, with both long gestations and highly invasive placentas. This circumstance results in an intimate association between maternal and fetal cells for nine months in normal pregnancy. Considering these features of human pregnancy, and the delicate balance between maternal immunocompetence and maternal–fetal tolerance that must be sustained throughout gestation, it is not surprising that pregnancy disorders, such as recurrent miscarriage (RM), preeclampsia (PE) and preterm birth (PTB), are common human conditions, all of which are associated with increased fetal or infant mortality and morbidity as well as long-term health consequences in the mother and her surviving infants^4–7^. Whether these deviations from normal gestational processes result from altered signaling between maternal and fetal cells that perturb implantation processes and/or disrupt maternal–fetal tolerance is unknown, largely due to the lack of good animal models of human pregnancy and the ethical challenges of studying cellular processes throughout human pregnancy.

A significant gap in knowledge of human pregnancy has been the composition of cells at the maternal–fetal interface (MFI) and differences between pregnancies with and without adverse outcomes. Recent single cell RNA sequencing (scRNA-seq) of first trimester^8–11^ or term^12–14^ placenta cells have filled some of this gap, but sample sizes were small (range 2–11 pregnancies) and only one included both term and preterm placentas^15^. Although these studies represented major steps forward in characterizing placenta cell types during the first trimester and at term, inferences were based solely on transcriptional signatures and provided little insight into potential mechanisms for spontaneous preterm birth. We provide here a complementary approach to address the latter.

To better understand the cellular changes that occur at the MFI following spontaneous labor at term (≥ 37 weeks gestation) and preterm (< 37 weeks gestation), we used mass cytometry to profile maternal and fetal cells located at the MFI at single-cell resolution from 27 term (16 laboring, 11 non-laboring) and 25 preterm (11 laboring, 14 non-laboring) pregnancies of women who self-identified as Black. This population was the focus of our study because they are a particularly high-risk group, with rates of preterm birth that are markedly higher than White women in the United States^16^. The resolution of mass cytometry allowed us to identify 25 immune cell types and six non-immune cell types based on cell surface markers. Our study revealed differences in maternal immune cell abundances between term laboring and non-laboring women and between maternal and fetal cell abundances at term and preterm. Together, our results suggest that the programmed cell death-1 (PD-1) and its ligand (PD-L1) pathway at the MFI may perturb the delicate balance between immune tolerance and rejection and contribute to spontaneous preterm labor.

## Results

### Overview

**Figure 1:**
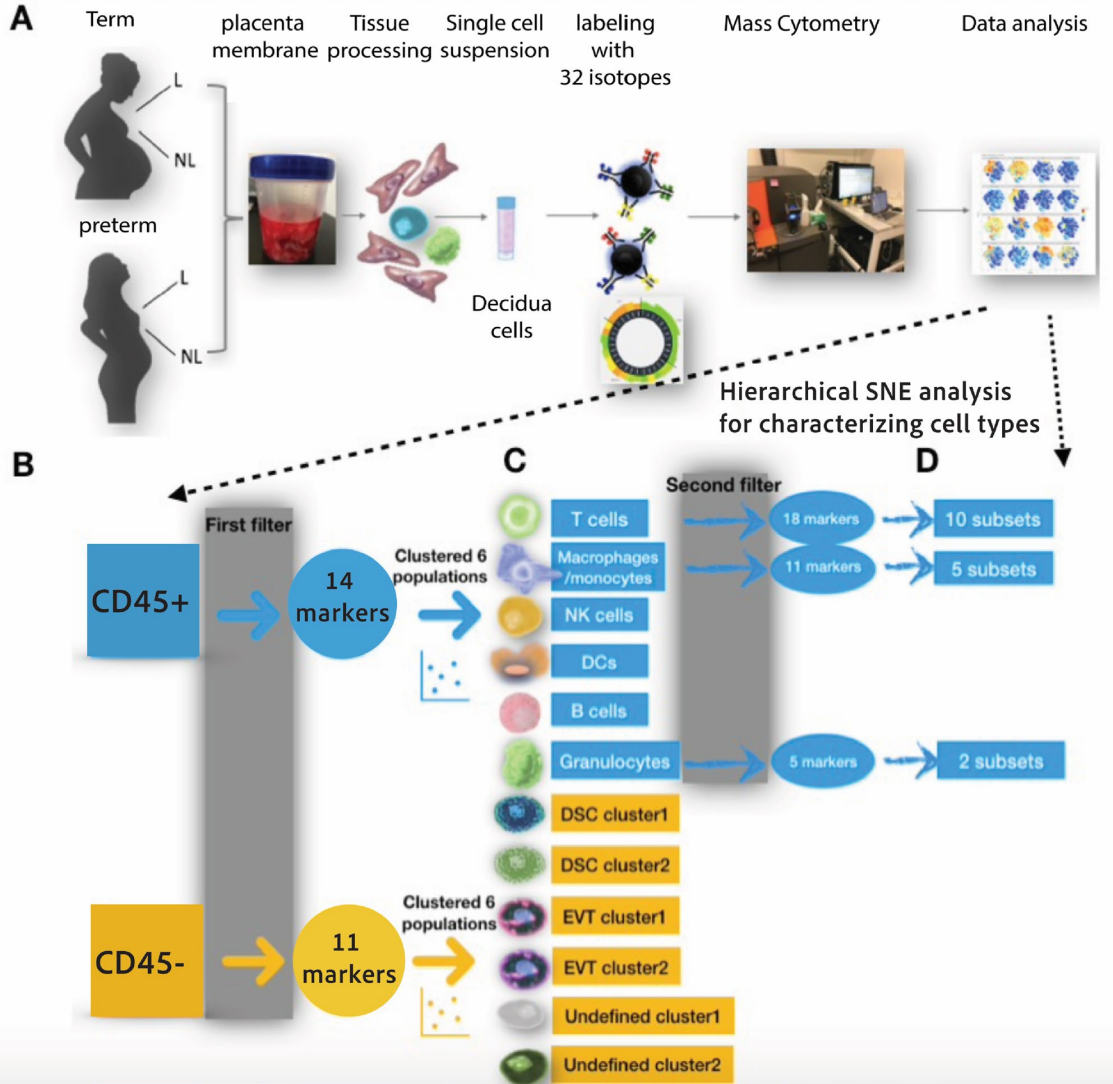
Experimental design and workflow. (**A**) Preparing decidual tissues for single cell analysis on the CyTOF platform. Our study included tissues from 16 term laboring, 12 term non-laboring, 11 preterm laboring, and 14 preterm non-laboring pregnancies (see Table 1). See Supplementary Methods for antibody staining with 32-antibody panel and CyTOF analyses. (B) After data processing and QC, the cells were first divided into CD45^+^ (immune) and CD45^-^ (non-immune) pools, then characterized using two additional filters for hierarchical SNE analysis. (**C**) The first filter identified six immune cell types and six non-immune cell populations by 14 and 11 markers, respectively. (**D**) The second filter identified 10 T cell subsets, five macrophage/monocyte subsets, and two granulocyte subsets. *L* laboring, *NL* non-laboring. *DCs* Dendritic cells, *DSC* Decidual stromal cell, *EVT* extravillous trophoblast. Undefined: No cell-surface markers to identify the cell type.

The main goal of this study was to use mass cytometry to characterize maternal and fetal cell types at the MFI by focusing on decidua tissues isolated from human term and preterm placentas. To understand the biological roles of cells at the MFI and discover distinct cell-type features as potential biomarkers of labor at term and preterm, we compared cell profiles between: (1) spontaneous term laboring (TL) and spontaneous preterm laboring (PL), (2) spontaneous TL and term non-laboring (TNL), (3) spontaneous PL and preterm non-laboring (PNL), and (4) TNL and PNL. An overview of our protocol for profiling single cells at the MFI is shown in Fig. 1A.

We included singleton pregnancies from women who were 18 years or older and who self-identified as Black. Membranes from 55 placentas were processed; 52 passed quality control after mass cytometry and were included in subsequent analyses. The final sample included placentas from 27 term and 25 preterm pregnancies; the 14 PNL placentas were from pregnancies with preeclampsia or eclampsia. The characteristics of the 55 women are shown in Table 1.

**Table 1:** Composition of the Study Sample.

Sample characteristics	Term		Preterm	
	Labor (TL)	Non-labor (TNL)	Labor (PL)	Non-labor (PNL)
Starting sample size	16	12	13	14
Sample size following QC	16	11	11	14
Mean maternal age ± SD (range) in years	26.1 ± 4.7 (19–35)	30.0 ± 2.9 (26–36)	24.6 ± 3.7 (19–29)	29.6 ± 5.2 (22–36)
Mean gestational age at delivery ± SD (range) in weeks	38.8 ± 0.9 (38–40)	39.0 ± 0.6 (38–40)	28.5 ± 4.5 (22–34)	30.4 ± 3.0 (26–34)
Infant sex (M:F)	4:12	5:6	6:5	9:5
Primigravidae (%)	31	0	18	0

All labor was spontaneous (see “Materials and methods”), all subjects self-identified as Black. Preterm non-laboring placentas were from women with preeclampsia or eclampsia undergoing cesarean section.

To enhance differences between the two groups, we selected term pregnancies from among women who delivered ≥ 38 + 0 weeks gestation and preterm pregnancies from among women who delivered between 22 + 0 to 34 + 6 weeks gestation We defined spontaneous labor as ≥ 6 regular uterine contractions in 60 min and either: (i) cervix ≥ 2 cm dilated or (ii) cervix ≥ 75% effaced. We excluded pregnancies with a fetal anomaly or a positive test for HIV, ZIKA, Hepatitis B, Hepatitis C or herpes. Based on pathological examinations of all preterm placentas, one was diagnosed with chorioamnionitis. However, the tissue sample from that placenta did not have increased neutrophils (a marker of infection) and did not appear as an outlier for any of the cells examined. Therefore, we retained this sample in our analyses.

### A human decidual cell atlas

To address the main goal of our study, we first identified the cell types present in decidual tissues from the four study groups using a mass cytometry panel of 32-metal isotope-tagged antibodies (Supplementary Table S1). This panel contained validated commercial antibodies that assess polarization of known immune populations that are also present in the periphery^17^ as well as antibodies that identify maternal and fetal non-immune cell populations at the MFI. We used Hierarchical Stochastic Neighbor Embedding (HSNE) for analyses of the hyperspectral satellite imaging data, as implemented in the Cytosplore platform, to cluster the data with a stepwise increase to the single-cell level (Fig. 1B–D)^18,19^. Abundances of cells in each subset for each of the four study groups and summary statistics for the analyses reported below are shown in Supplementary Tables 3–5.

**Figure 2:**
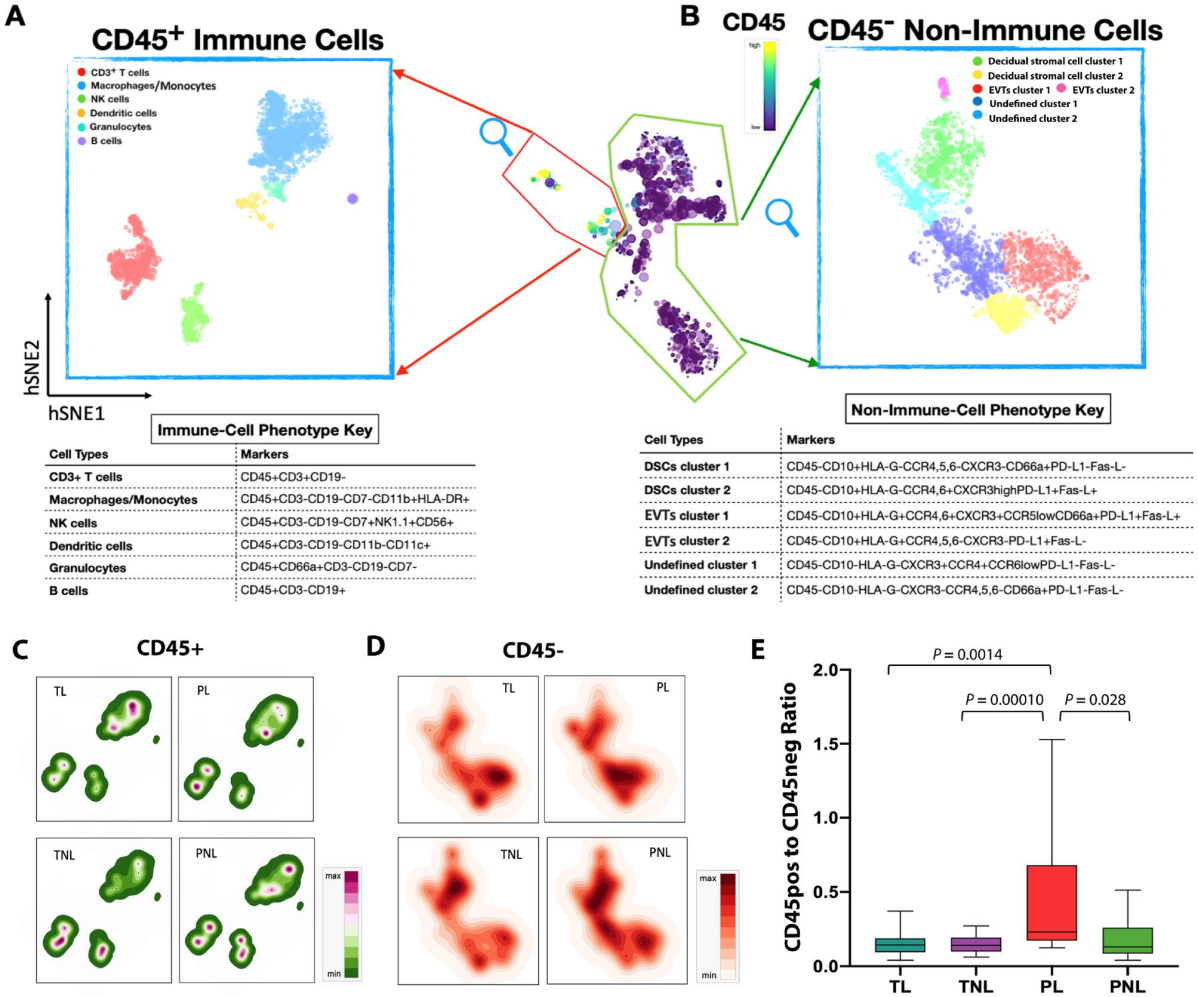
Twelve distinct cell lineages were identified following the first filter. Cells were clustered as CD45^+^ (immune) cells (**A**) and CD45^-^ (non-immune) cells (**B**). Six major immune populations (A) and six non-immune cell populations (**B**) were color coded by Cytosplore^+HSNE^. hSNE visualization of the CD45 + (**C**) and CD45- cells (**D**) as a density plot for each of the four groups is shown. Each point is an individual subject. The cells were grouped based on the Euclidian distances of marker expression and populations were assigned based on the markers. The representative cells were found by a weighted k-nearest neighbor (kNN) graph and served as landmarks. The size of each point indicates the area of influence (AoI) of the landmarks. Scatterplots of marker expression are shown in Supplementary Fig. S1. The ratios of CD45pos to CD45neg cells by study group (**E**).

We first divided the data into the immune (CD45^+^) and non-immune (CD45^-^) compartments (Figs. 1B and 2), and then applied additional filters for deeper clustering. As the first filter, we used 14 cell-surface markers to define six major immune cell types and 11 cell-surface markers to define six non-immune cell populations (Fig. 1C; Supplementary Fig. 1).

We next used 18, 11, and 5 cell-surface markers as a second filter for clustering of T cells, macrophages/monocytes, and granulocytes subsets, respectively (Fig. 1D, Supplementary Table 2). We identified 31 cell populations in human decidual tissues, with 2 broad subsets of CD45 + and CD45- cells comprised of 6 immune cells and 6 non-immune cells after the first filter (Fig. 2A,B). The proportions of CD45 + and CD45- cells for the four study groups are shown in Fig. 2C,D and Supplementary Fig. 2. Whereas the ratios of CD45 + cells to CD45- cells were similar in TL (median = 0.144), TNL (median = 0.142), and PNL (median = 0.130) (Robust Rank-Order test, *P* > 0.95 in pairwise comparisons), the ratio was significantly higher in PL (median = 0.230; Robust Rank-Order test, *P* = 0.0014, 0.0001, 0.028 compared TL, TNL, and PNL, respectively) (Fig. 2E). These combined data suggest that fluctuations in the ratios of CD45 + to CD45 − cells are not a feature of labor vs. non-labor at term or between non-labor at term or preterm. Rather, the increased ratio in PTL compared to the other three groups suggests that spontaneous preterm labor may be characterized by shifts in cell composition that results from increased proportions of immune cells and decreased proportions of non-immune cells at the MFI.

**Figure 3:**
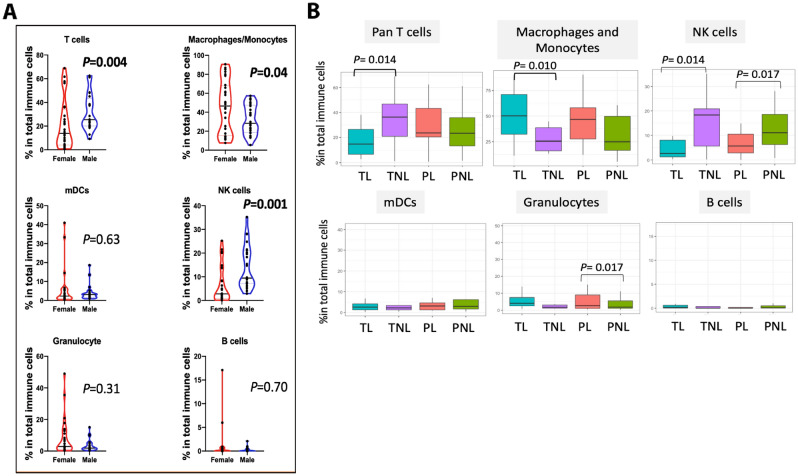
Six immune cell clusters at the maternal–fetal interface. (**A**) Violin plots showing cell abundance by infant sex. *P*-values are shown for pairwise comparisons with *P* ≤ 0.05. Each point is an individual; red denotes a female infant and blue a male infant. (**B**) Boxplots showing relative abundances of the six immune cell populations in the four study groups**.** The horizontal lines show the median percent of total immune cells, and the vertical lines show the interquartile ranges. Pairwise differences between groups at nominal *P* ≤ 0.05 are shown. Test statistics (Robust Rank-Order test) with *P*-values and descriptive statistics are shown in Supplementary Tables S3 and S5, respectively.

To specifically characterize the immune cell lineages, 3,000 CD45^+^ cells from each sample were randomly extracted and pooled together for cell-type profiling (156,000 CD45^+^ cells in total). Using different combinations of immune-cell surface markers (Supplementary Table S2), we identified six major innate and adaptive immune populations at the MFI: CD3^+^ T cells (pan T cells), macrophage/monocytes, NK cells, dendritic cells (mDCs), granulocytes and B cells (Fig. 2A). Macrophages/monocytes and T cells were the most abundant cell types, followed by NK cells, DCs, granulocytes, and B cells. The NK cell, DCs, and B cell populations were not further filtered. Two granulocyte subsets were identified (eosinophils and neutrophils) but both were sparse and not further investigated (Supplementary Fig. 3). The distributions of the abundances of these six cell types among the four groups are shown in Fig. 2C. None of the major immune cell types defined on the first filter differed among the four study groups after considered the potential effect of fetal sex on the relative abundances, except possibly granulocytes (Fig. 3; Table 1; Supplementary Results and Supplementary Fig. 4). The increased abundances of T cells and NK cells in pregnancies with male infants were seen in both preterm and term, as well as laboring and in non-laboring, pregnancies (Supplementary Table 6).

**Figure 4:**
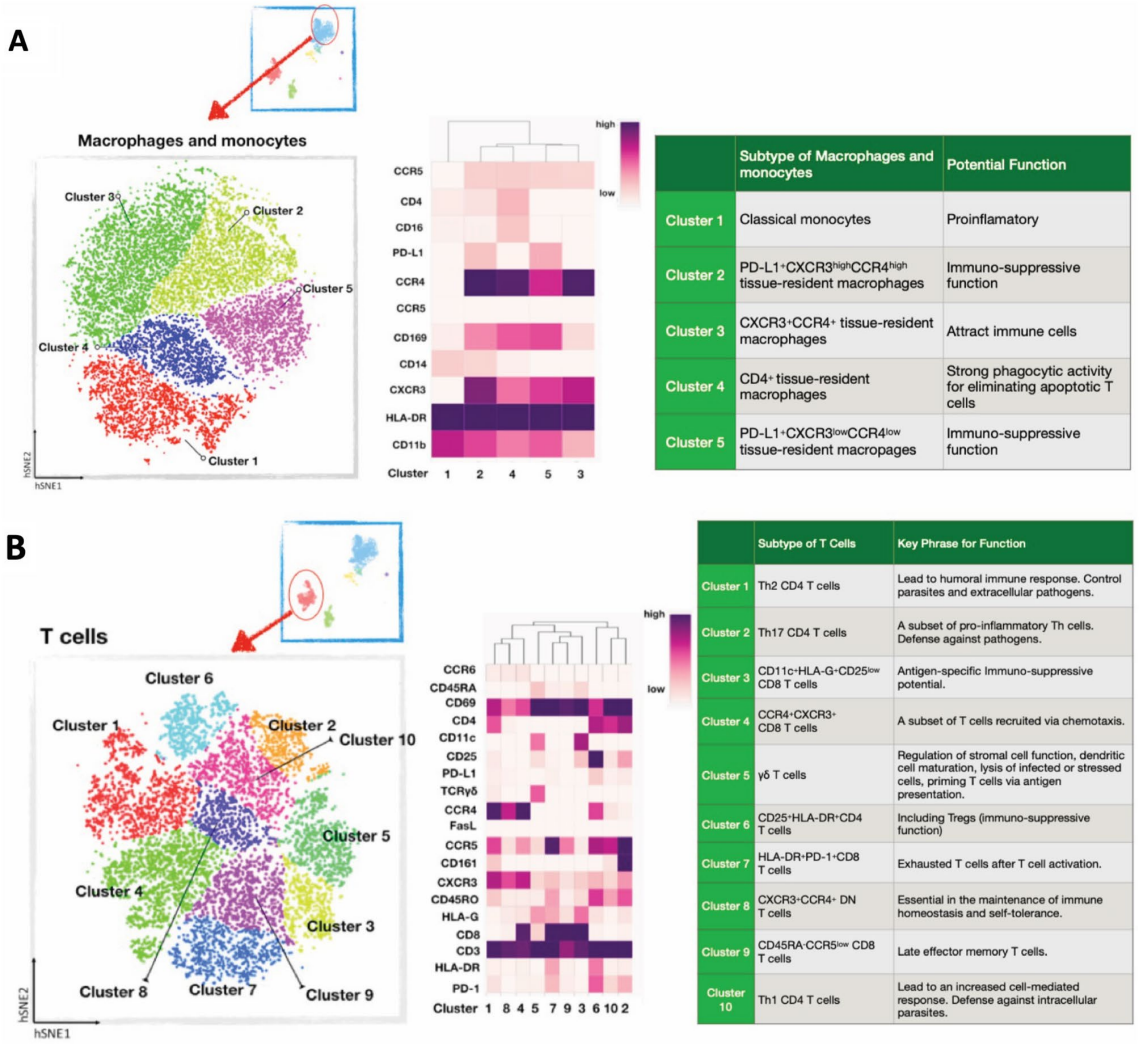
Deep profiling of T cells and macrophages/monocytes at the maternal–fetal interface. 10 clusters of T cells based on the expression of 19 T-cell surface markers (see Figs. 1, 2) (**A**) and five clusters of macrophages/monocytes based on the expression of 11 cell surface markers (**B**). Each point represents a cell. The heat map views (center panels) are ordered by similarity as shown by the dendrogram above the heatmaps. The tables (right panels) describe the cell-subtype assignments and their potential cellular functions. Descriptive statistics of these data are shown in Supplementary Table S5.

Using the cell-surface markers defined on the second filter of immune cells (Fig. 1D), we identified five macrophage/monocyte and 10 T cell subsets (Fig. 4; Supplementary Fig. 5; Supplementary Tables 7, 8). Cells in macrophage/monocyte cluster 2 were more abundant in PL compared to PNL (z = 2.63, *P* = 0.0083) and in TNL compared to PNL (z = 3.03, *P* = 0.0024), while cells in cluster 5 were more abundant in TL compared to TNL (z = 2.02; *P* = 0.043) (Supplementary Fig. 5). The macrophage/monocyte clusters 2 and 5 cells both had an immune-suppressive phenotype (Fig. 4A), defined as PD-L1^+^CXCR3^high^CCR4^high^ and PD-L1^+^ CXCR3^low^CCR4^low^, respectively, suggesting an increase in these cell types during labor. The observation of increased proportions of myeloid cells in TL compared to TNL is consistent with studies in mice^20,21^ and in women^22^. The T cells are discussed in more detail below.

The relationships between macrophage/monocyte cell clusters and gestational age, fetal sex, and labor are described in Supplementary Results, Supplementary Figs. 6, 7, and Supplementary Tables 3 and 5.

### T cell subsets at the MFI differ between term and preterm woman

**Figure 5:**
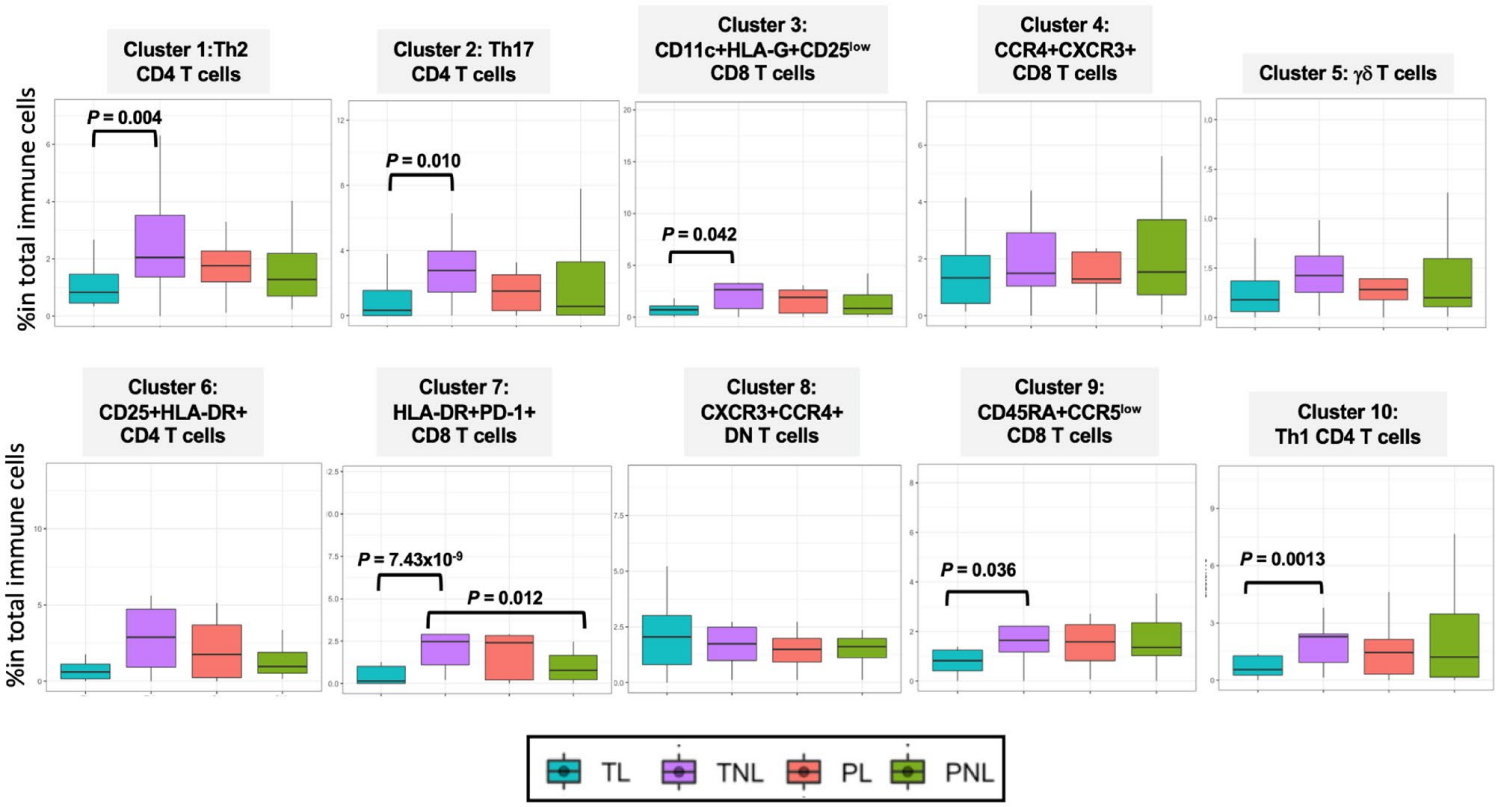
Distributions of 10 T-cell subtypes at the maternal–fetal interface in each study group. In each boxplot, the horizontal lines show the median percent of total immune cells and the vertical lines show the interquartile ranges. Pairwise differences between four groups at nominal *P* ≤ 0.05 are shown (Robust Rank-Order test). Summary and descriptive statistics of these data are shown in Supplementary Tables S3 and S5, respectively. T cell abundances by sex and study group are shown in Supplementary Fig. 8.

Ten phenotypically distinct subsets of T cells were present at the MFI (Fig. 4B; Supplementary Table 7). None of the 10 subsets differed by gestational age (Supplementary Fig. 8), but the abundances of T cells in clusters 1, 3, 4, 5, 6, 9 and 10 were higher in pregnancies with male compared to female infants (Supplementary Fig. 9). Cells in six clusters were less abundant in TL compared to TNL at *P* ≤ 0.05: clusters 1 (*P* = 0.0041), 2 (*P* = 0.0098), 3 (*P* = 0.042), 7 (*P* = 7.43 × 10^–9^), 9 *(P* = 0.036), 10 *(P* = 0.0013) (Fig. 5; Supplementary Tables 3 and 5). However, these differences may be due to sex ratio imbalances between TL and TNL (Table 1, Supplementary Results, Supplementary Tables 3 and 5). The phenotype of cluster 7 is one of activated T cells that express the programmed cell death (PD-1) receptor. PD-1 binds to its receptor, PD-L1, to suppress adaptive immune responses and maintain immune balance^23^. The lower abundance of these cells in TL, and the relative higher abundance of these cells in PL compared to TL, raised the possibility that cells at the MFI with inhibitory signals from PD-L1 may also differ between term and preterm labor. Thus, we next investigated the abundances of PD-L1^+^ non-immune cells in this niche.

### CD45^–^ non-immune cell subsets at the MFI in term and preterm pregnancies

**Figure 6:**
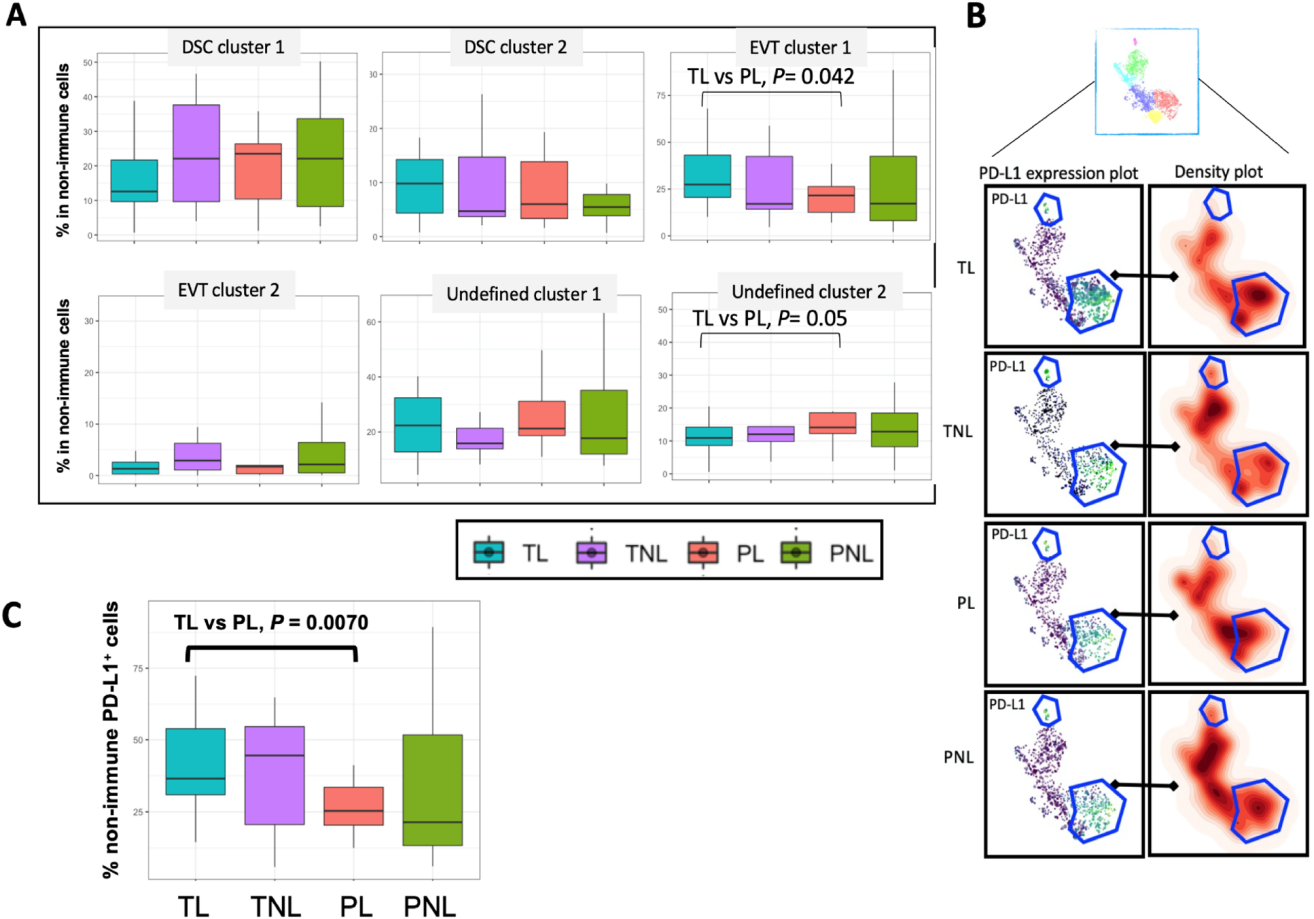
PD-L1^+^ non-immune cells at the maternal–fetal interface. (**A**) Box plots showing the distribution of six clusters of non-immune cells. The horizontal lines show the median percent of total non-immune cells and the vertical lines show the interquartile ranges. Pairwise differences between groups at nominal *P* ≤ 0.05 are shown (Robust Rank-Order test). (**B**) hSNE visualization of PD-L1^+^ non-immune cell lineages as a density plot for each of the four groups. Each point is an individual subject. (**C**) Box plots of PD-L1^+^ non-immune cells by study group. The horizontal lines show the median percent of total non-immune cells, and the vertical lines show the interquartile ranges. Pairwise differences between groups at *P* ≤ 0.05 are shown (Robust Rank-Order test). Summary and descriptive statistics of these data are shown in Supplementary Tables S4 and S5, respectively.

To explore the non-immune cell compartment, 10,000 CD45^-^ cells from each sample were randomly extracted and pooled together for cell-type profiling (520,000 CD45^-^ cells in total). Six major non-immune cell populations were clustered by 11 markers, including CD10, HLA-G, and PD-L1 (Fig. 2B), defining two clusters of decidual stromal cells (DSCs) and two clusters of EVTs. Two additional unknown cell clusters were defined as CD10 negative and HLA-G negative, and may represent the novel endothelial cells described in scRNA-seq studies^15^. Of the six major non-immune cell clusters, cells in EVT cluster 1 (CD45^-^CD10^+^HLA-G^+^CCR4,6^+^CXCR3^+^CCR5^l^^ow^CD66a^+^PD-L1^+^Fas-L^+^) were less abundant in PL compared to TL (*P* = 0.042) and cells in undefined cluster 2 (CD45^-^CD10^-^HLA-G^-^CXCR3^-^CCR4,5,6^-^CD66a^+^PD-L1^-^Fas-L^+^) were more abundant in PL compared to TL (*P* = 0.054). (Fig. 6A, Supplementary Tables 4, 5). These results suggested that PD-L1 + non-immune cells at the MFI are deficient in PL pregnancies compared to PL.

The relationships between non-immune cell types, infant sex, gestational age, and labor are described in Supplementary Results, Supplementary Figs. 10 and 11, and Supplementary Tables 4, 5.

### PD-L1^+^ non-immune cells are less abundant in preterm labor

The significantly lower abundances of HLA-DR^+^PD-1^+^ CD8 T cells (T cell cluster 7) in TL compared to TNL (*P* = 7.43 × 10^–9^) and a trend toward lower abundances of these cells in TL compared to PL (*P* = 0.069), as well as more abundant PD-L1^+^ EVT cluster 1 cells in TL compared to PL (*P* = 0.042) suggested a potential role of the PD-1/PD-L1 pathway in preterm birth. To further explore this possibility, we focused on the two clusters of PD-L1^+^ EVTs and one cluster of PD-L1^+^ DSCs among the non-immune cells (Fig. 6). The PD-L1^+^ cells were similar in abundance between TL and TNL (*P* = 0.80) and between PL and PNL (*P* = 0.93) (Fig. 6A; Supplementary Tables 4, 5) but were more abundant in TL compared to PL (*P* = 0.0070), consistent with the observation of fewer PD-1^+^ CD8 T cells in TL compared to PL (Fig. 5).

Cells from all three PD-L1^+^ clusters (DSC cluster 2, EVT cluster 1, and EVT cluster 2) contributed to these differences (Fig. 6B). Further examination of the relationship between PD-L1^+^ non-immune cell abundances and gestational age (Supplementary Fig. 12) suggested that the abundances of PD-L1^+^ non-immune cells at the MFI are not influenced by gestational age per se but rather may be a feature of labor at term (Supplementary Methods). Infant sex was not likely contributing to the differences in abundances of PD-LI^+^ non-immune cells between TL and PL (Supplementary Methods and Supplementary Fig. 12). Together with the previous observations of increased abundance of PD-1^+^ T cells (T cell cluster 7) in PL compared to TL (Supplementary Table 5), these data suggested that preterm labor may be associated with a lack of suppression of activated T cells at the MFI. However, whether this is due to intrinsic differences in the regulation of PD-LI^+^ cells and causally related to preterm birth cannot be determined in this observational study. Therefore, we used a cell culture model of decidua-derived mesenchymal stromal cells from term and preterm placentas to further investigate this possibility.

### Decreased transcription of PD-L1 in cultured stromal cells from preterm placentas

**Figure 7:**
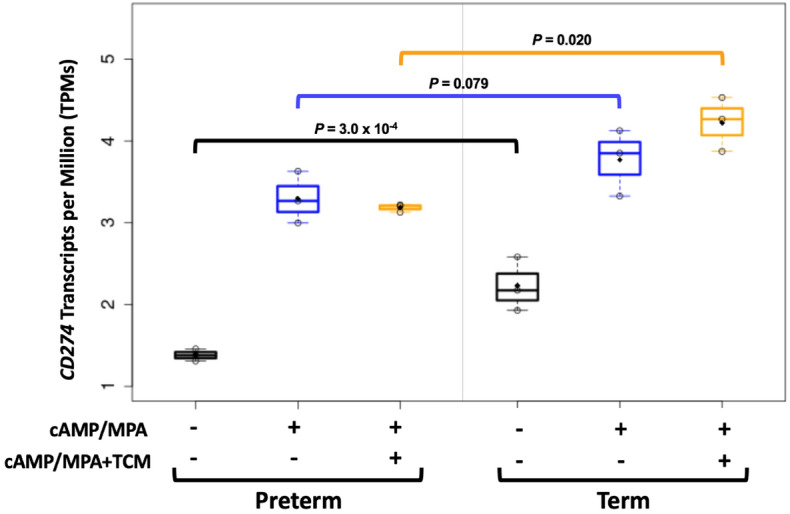
Transcript levels of the gene encoding PD-L1, *CD274*, in cultured mesenchymal stromal cells from term and preterm placentas. *CD274* transcript levels were measured in stromal cells from three term and three preterm placentas after culturing in media alone, in decidualization media (cAMP/MPA), and in decidualization media with trophoblast conditioned media (cAMP/MPA + TCM) (Materials and methods). P-values from Wald test.

We isolated stromal cells from placental membranes and established primary cell lines from three spontaneous TL and three spontaneous PL placentas that were not included in the mass cytometry studies. To assess transcriptional differences in *CD274*, the gene encoding PD-L1, we purified RNA from cells cultured in three conditions: media alone, media plus cAMP and MPA (medroxyprogesterone acetate) to induce decidualization and mimic pregnancy conditions, and decidualization media plus trophoblast conditioned media (TCM) that contains fetal-derived signaling molecules secreted by trophoblasts (Materials and methods). After standard processing and QC (Materials and methods and Sakabe et al.^24^), RNA sequencing data from the three replicates of each sample were pooled. Transcript levels of *CD274* were strikingly lower in decidua cells from all three preterm placentas compared to decidua cells from three term placentas when cultured in media alone (Wald test *P* = 3.0 × 10^–4^) (Fig. 7). Transcript levels increased in all samples in response to decidualization treatment, reducing differences between preterm and term cells (Wald test *P* = 0.079), but levels further increased in response to TCM in the term cells only, restoring differences between cells from PTL and TL (Wald test *P* = 0.020). Other checkpoint genes were either very lowly expressed (*TIM3*, *LAG3*; < 1 transcript per 1000 in all samples) or not expressed (*CTLA4*; 0 transcripts per 1000 in nearly all samples) in all three conditions (data not shown). These results suggest intrinsic differences in the constitutive expression of *CD274* in stromal cells and in the regulation of *CD274* by TCM signaling molecules in decidualized cells from preterm compared to term placentas.

## Discussion

Many previous studies have suggested that the PD-1/PD-L1 immune checkpoint pathway plays a critical role in maintaining immune tolerance at the MFI^25–38^. In mice, blocking PD-L1 results in increased rejection of allogeneic, but not syngeneic, conceptuses^36,37^. In humans, evidence for a role in recurrent miscarriage (RM) is supported by studies showing reduced abundance of PD-L1^+^ cells or expression of its gene in first trimester trophoblasts from pregnancies of women with a history of RM compared to first trimester trophoblasts from pregnancies of women without prior miscarriages^34,38^. In contrast, PD-1^+^ and PD-L1^+^ cells were more abundant in term placentas from women with PE compared to placentas from women without PE at term^33^, and PD-L1^+^ immune cells were more abundant in the periphery of women with early onset (< 34 weeks gestation) PE compared to term non-PE controls^29^. Indirect evidence for a role of PD-L1 in preterm birth was provided by a mouse model of LPS-induced preterm labor: erythropoietin both prevented preterm labor and upregulated placental expression of PD-L1^39^. However, to our knowledge, no studies to date have examined a potential role for the PD-1/PD-L1 pathway in spontaneous preterm birth in humans. Our study provides several lines of evidence supporting a role for this important pathway in PTB.

Using an unbiased approach to characterize maternal and fetal cells at the MFI from Black women, who are at high risk for PTB^16^, we identified both PD-1^+^ CD8 T cells and PD-L1^+^ non-immune cells as the most significantly associated cell types with spontaneous term and preterm birth, respectively. The median relative abundance of PD-1^+^ CD8 T cells (cluster 7) at the MFI was 0.14 in TL pregnancies compared to 2.47 in TNL (*P* = 7.43 × 10^–8^) and 2.40 in PL (*P* = 0.069) pregnancies (Supplementary Table 5), differences that could not be attributed to sex ratio imbalances between the groups (Supplementary Fig. 9). These observations led us to suggest that PD-L1^+^ cells, which suppress expression of PD-1^+^ cells, would be more abundant in cells at the MFI in TL pregnancies compared to TNL. In line with our suggestion, PD-L1^+^ macrophages in cluster 5 cells (PD-L1^+^CXCR3^low^CCR4^low^) were more abundant in TL compared to TNL (*P* = 0.043) (Supplementary Table 3). This immunosuppressive phenotype was unexpected in TL because the onset of labor, or parturition, has been proposed to be an inflammatory process^22,40,41^. For example, in a study of peripheral T cell responses to antigen in the periphery during pregnancy^42^, Shah and colleagues observed a selective dampening of primary CD8 T cell immune responses during the second trimester that were coincident with increased expression of CD8 PD-L1^+^ effector memory cells. This pattern was reversed in the third trimester leading the authors to suggest that a relaxation of immune tolerance occurs prior to the onset of labor at term, a suggestion that is inconsistent with the results of our study of cells at the MFI. In contrast, a recent longitudinal study of immune trajectories in the periphery during pregnancy reported a dampening of systemic inflammatory events with approaching labor^43^, more in line with the results of our study of cells at the MFI. The novel observation in our study of increased ratios of CD45 + to CD45- cells in PL (Fig. 2E and Supplementary Fig. 2) further suggested a more immunologically activated state in preterm labor compared to both term labor and non-labor at term or preterm.

Among the non-immune cells at the MFI, PD-L1^+^ cells (DSC cluster 2 and EVT clusters 1 and 2) were significantly more abundant in TL compared to PL (*P* = 0.0070). This finding is consistent with previous reports of reduced expression of PD-L1^+^ cells and expression of its gene in early pregnancy trophoblast cells from women with a history of RM compared to non-RM women^34,38^ and raises the possibility that spontaneous preterm labor and RM have a shared etiology related to intrinsic deficiencies in PD-L1 expression on non-immune cells at the MFI. We further explored the hypothesis of intrinsic defects in PD-L1 expression contributing to spontaneous preterm labor using a cell culture model of primary mesenchymal stromal cell decidualization and response to trophoblast secreted factors in TCM. Considering three culture conditions, we showed that expression of *CD274*, the gene encoding PD-L1, was significantly less expressed in cells from preterm pregnancies compared to term pregnancies (*P* = 3.0 × 10^–4^). Expression was increased after decidualization treatment in cells from both preterm and term pregnancies, however, the expression of this gene was further increased in response to TCM in cells from term pregnancies whereas cells from preterm pregnancies were largely unresponsive to fetal signaling molecules in TCM (Fig. 6). Reduced expression of *CD274* in mesenchymal stromal cells from preterm pregnancies in the untreated state and in conditions that mimic pregnancy (decidualization plus trophoblast-secreted molecules) suggests a fundamental defect in these cells in spontaneous preterm labor. These results provide independent evidence that non-immune cells at the MFI in preterm pregnancies have lower *CD274* expression in conditions that mimic pregnancy.

An earlier study by Enninga and colleagues measured soluble (s)PD-L1 expression in blood from women before, during, and after pregnancy^27^. They did not observe any differences in sPD-L1 levels between pregnancies with male or female fetuses, similar to the PD-L1^+^ cells at the MFI in our study. Mean levels of sPD-L1 increased between 8 and 35 weeks gestation; by 15 weeks of gestation, levels were significantly higher than those in non-pregnant control women and remained so through delivery. We did not observe correlations between gestational age at delivery and abundances of PD-L1^+^ non-immune cells at the MFI in the non-laboring pregnancies (Supplementary Fig. 12B). However, because the non-laboring preterm pregnancies were all in women with severe PE, a greater abundance of PD-L1^+^ non-immune cells in PE pregnancies^33^ may have masked this effect. Indeed, the abundances of PD-L1^+^ non-immune cells were remarkably similar between laboring and non-laboring term pregnancies (term labor vs. term non-labor, *P* = 0.41). In the laboring pregnancies, we observed a correlation between PD-L1^+^ cell abundance and gestational age that did not reach statistical significance (*P* = 0.06) (Supplementary Fig. 12), but this trend was driven largely by the greater abundance of PD-L1^+^ non-immune cells in term pregnancies. There was little evidence for an increasing trend between 20 and 35 weeks. Without more data points, including some before 20 weeks, it is not possible to further interpret these data, but they are consistent with the gene expression studies indicating an intrinsic deficiency of PD-L1 expression in PTB.

There are limitations to our study that warrant careful interpretation of our results. First, although our sample sizes were larger than previous single cell studies of placenta cells^8–15^, they were still relatively small, especially considering the amount of variation observed for some cell types between individuals and between those with male or female infants. As a result, we did not apply multiple testing corrections and relied on nominal levels of significance (*P* ≤ 0.05) to identify potentially interesting findings. Thus, our results should be considered suggestive, requiring replication or functional validation in independent studies. Second, the TL and PNL samples had significant skewed sex ratios. This could be attributed to random fluctuations in small samples or, possibly, to biological causes. Regardless, these imbalances made it impossible to disentangle sex effects from true immune cell effects for some of our results. Third, we focused on phenotyping major cell populations at the MFI based on surface markers. Therefore, we were not able to identify cell populations that require intracellular staining or lack known protein-level markers. For example, we identified EVTs based on HLA-G expression but could not specifically target villous cytotrophoblast or syncytiotrophoblast cells. Similarly, we could not differentiate between M1 and M2 macrophages or classify regulatory T cells (Tregs) that differ based on Foxp3 expression. Despite these limitations, however, our study revealed novel findings that further our understanding of spontaneous preterm labor.

Although the mechanisms underlying this process at term and preterm are still largely unknown^2^, it is likely that both maternal and fetal signals contribute to the tolerogenic state that exists between mother and fetus during pregnancy, and to the relaxing of tolerance preceding or during parturition. The PD-1/PD-L1 pathway has been considered an important player in maintaining tolerance during pregnancy, but its role in relaxing tolerance at the onset of spontaneous labor has not been demonstrated. The results of our study suggest that lessening of PD-L1-mediated suppression of activated T cells at the MFI may not be a likely mechanism for parturition at term. We observed both low levels of PD-1^+^ CD8 T cells and high levels of PD-L1^+^ macrophages and PD-L1^+^ non-immune cells associated with spontaneous term labor, consistent with a suppressive state. However, we presented convergent support for the idea that spontaneous preterm labor may be attributed to, at least in part, impaired *CD274* expression and deficiencies of PD-L1^+^ maternal and fetal non-immune cells at the MFI. This deficiency could inhibit the suppression of activated PD1^+^ immune cells and trigger preterm labor through its effects on the regulatory landscape at the MFI^28,33–35,39^. Taken together with published studies, we suggest that mechanisms triggering spontaneous labor differ at term and preterm, with spontaneous preterm labor due to deficient expression of PD-L1, possibly a shared etiology with some first-trimester miscarriages.

## Materials and methods

### Patient recruitment and clinical characteristics for this study

Decidual membranes were collected from placentas of women delivered at Chicago Lying-In Hospital at the University of Chicago or Prentice Women’s Hospital at Northwestern University (Table 1). Spontaneous labor was defined as having six or more regular uterine contractions in 60 min, and either the cervix dilated ≥ 2 cm or ≥ 75% effaced, prior to the rupture of membranes. All patients in PNL group had preeclampsia or eclampsia. One of the placentas had evidence of clinical chorioamnionitis based on pathological exam of all preterm placentas, but no evidence of neutrophils or granulocytes in the decidual membranes. All patients in the TL and TNL group had healthy, uncomplicated pregnancies. These studies were approved by the Institutional Review Boards at the University of Chicago and Northwestern University and all participants signed an informed consent. All research conformed to the Declaration of Helsinki.

### Human decidual tissues collection and cell isolation

1–2 5 cm^2^ pieces of decidua membrane were sampled within 1 h of delivery from the basal plate of the placenta at a site that was distant from the rupture site. Pieces were immediately placed in Dulbecco’s modified Eagle’s medium (DMEM)-Ham’s F12 media with 10%FBS and Pen/Strep and then stored at 4 °C until processing, which occurred within 48 h of delivery. The amnion and blood clots were removed from the membranes, which were then placed with the decidua side up in a petri-dish. The decidual tissue was then gently scraped away from the chorion and the chorion and decidua were placed into separate petri dishes. Each piece was then minced into small pieces about 1 mm^3^, rinsed with D-PBS, and placed into a 50 ml falcon tube with 20 ml digestion buffer containing collagenase, hyaluronidase, and DNase. The tubes were incubated in a shaking water bath (150 rpm, 37 °C) for 30 min. After incubation, the cell digest supernatant was filtered through a 70um nylon filter into sterile 50 ml conicals. The filtrate was then centrifuged at 1200 rpm for 10 min to collect the dissociated cells, which were then washed in RPMI. The digestion steps (incubation through RPMI wash) were repeated one additional time and then resuspended in culture medium. Cells from the chorion and from the decidua underwent red blood cell removal and were then counted by trypan blue exclusion. The remaining cells were frozen separately in 90% FBS/10% DMSO freezing media (Gibco). Only the cells from the decidua were used for this study.

#### Mass cytometry and data generation

Single-cell mass cytometry was applied to the decidua cells. The details of panel design, antibody titration, quality control, batch effect testing, and cell staining are described in Supplementary methods.

After cell staining and preparation, samples were collected on Fluidigm Helios™ mass cytometer in Cytometry and Antibody Technology Core Facility at University of Chicago. The raw data were plotted by time vs beads to monitor the uniformity of the data and signal over time. The generated .FCS files were normalized by the Normalizer (https://github.com/nolanlab/bead-normalization/releases) in the MATLAB Compiler Runtime (MCR) environment^44^. Here, we used MCR Release R2013b (8.2) for Mac OSX. Then the normalized data with bead removal were analyzed by FlowJo v10.0.7, Cytofkit^45^, and Cytosplore^18,19^ software. Data analysis and visualization procedures are described in Supplementary methods.

### Pairwise comparisons of cell abundances between study groups

Abundances of immune and non-immune cell subsets were compared between TL and PL, TL and TNL, PL and PNL, and TNL and PNL, and between pregnancies with male and female infants, using the Robust Rank-Order test in RStudio (v.1.0.143) (v.3.5.1), package “trend”, usage “rrod.test(x, y, alternative = c(“two.sided”)”. For analyses of each cell subset type, outliers were identified by boxplots in r package “ggplot2” and “readr” as those with values that were greater than 1.5 times the interquartile range above the third quartile or less than 1.5 times the interquartile range below the first quartile. Any samples designated as outliers were removed using the ‘remove outliers’ command in RStudio (v.1.0.143) R (v.3.5.1) boxplot function: “x[!x %in% boxplot.stats(x)$out]” in analyses of that specific cell subset type. Correlations between gestational age and cell abundances were examined using Pearson Correlation test as implemented in Prism 9.

#### Preparation of trophoblast conditioned media (TCM), cell culture and gene expression studies

Placentas were collected from nine Black women (≥ 18 years old) who delivered at term (≥ 37 weeks) following spontaneous labor with vaginal delivery (n = 3) or non-labor with c-section (n = 6) of singleton pregnancies. These women were unrelated to those used to establish the six cell lines described above. Tissue samples were collected as described above, kept at 4 °C and processed within 24 h of tissue collection. Primary cytotrophoblast (CTB) cells were isolated from placental membranes as previously described^46^. Cultured primary CTB cells were washed and 10 ml of Control Media (1 × phenol red-free high-glucose DMEM, 2% CS-FBS, 2 mM L-glutamine) were added to each plate. After 48 h, the conditioned media was removed and stored at − 80 °C. The TCM from 9 placentas were thawed, pooled and 0.5 mM 8-Br-cAMP and 1 μM MPA Progesterone (MPA) were added to make the conditioned-decidualization media.

Mesenchymal stromal cells lines (MSCs) obtained from term (n = 3) and preterm placentas (n = 3) were plated (5 × 10^5^ cells per 60- mm plate) and grown for 2 days in decidualization media (1 × phenol red-free high-glucose DMEM, 2% CS-FBS, 2 mM % l-glutamine, 0.5 mM 8-Br-cAMP and 1 μM MPA). After 48 h, cells were treated with TCM + decidualization media or decidualization media alone for another 48 h. At the end of the incubation period, cells were harvested and pelleted for RNA isolation. Each experiment was performed in triplicate.

Protocols for RNA-sequencing, quality control steps and analyses were described in our earlier study^24^. Briefly, normalized read counts of the *CD274* transcript were compared between cells from term and preterm placentas in each of the three conditions after correcting for unwanted variation^47^.

## Supplementary Information

Supplementary Information.

## Data Availability

All CyTOF data generated or analyzed during this study are available at https://www.immport.org/shared/study/SDY2075. All software and algorithms used to process data are shown in Supplementary Table 9.
