# Supplementary material for: Single cell profiling at the maternal–fetal interface reveals a deficiency of PD-L1+ non-immune cells in human spontaneous preterm labor
Source: Sci Rep. 2023 May 16;13:7903. doi: 10.1038/s41598-023-35051-5 (PMC10188528; doi:10.1038/s41598-023-35051-5)
Supplement: Supplementary file 1 — Supplementary Information. [file 41598_2023_35051_MOESM1_ESM.pdf]

Supplementary Materials

Single Cell Profiling at the Maternal-Fetal Interface Reveals a Deficiency of PD-L1+ Non-Immune Cells in Human Spontaneous Preterm Labor

Xiao Liu[1], Ivy Aneas[1], Noboru Sakabe[1], Rebecca Anderson[1], Christine Billstrand[1], Cristina Paz[1], Harjot Kaur[1], Brian Furner[2], Seong Choi[2], Adriana Y. Prichina[1], Elizabeth Ann L. Enninga[3], Haidong Dong[4], Amy Murtha[5], Gregory E. Crawford[6], John A. Kessler[7], William Grobman[8], Marcelo A. Nobrega[1], Sarosh Rana[9], Carole Ober[1]

Departments of Human Genetics[1], Center for Research Informatics[2], and Obstetrics and Gynecology[9], University of Chicago, Chicago, IL
Departments of Obstetrics and Gynecology[3] and Immunology[4], Mayo Clinic, Rochester, MN
Department of Obstetrics and Gynecology[5], Duke University Health Systems, Durham, NC
Department of Pediatrics and Center for Genomics and Computational Biology[6], Duke University, Durham, NC
Departments of Neurology and Institute for Stem Cell Medicine[7] and Obstetrics and Gynecology[8], Feinberg School of Medicine, Northwestern University, Chicago, IL

Table of Contents

**Supplementary Methods**	**Page**
Panel Design and Antibody Titration	5
Protocol Modifications for Cell Preparation Before Antibody Staining	5
Cell and Bead Staining	6
Sample Quality Control	6
Testing of Signal Spillover	6
Testing for Batch Effects	7
CyTOF Data Analysis and Visualization	

**Supplementary Results**	**Page**
Effects of gestational age and sex on cell distributions	11

**Supplementary Tables**	**Page**
Table S1. CyTOF panel	15
Table S2. Cell surface markers on the second filter	16
Table S3. Immune cell z-scores and *P*-values for pairwise comparisons	17
Table S4. Non-immune cell z-scores and *P*-values for pairwise comparisons	18
Table S5. Distributional statistics for immune and non-immune cells	19
Table S6. T cell and NK cell abundances by labor type and term/preterm	20
Table S6. T cell subset markers	21
Table S7. Macrophage/monocyte subset markers	21
Table S7. Software and algorithms	23

**Supplementary Figures**	**Page**
Figure S1. H-SNE maps of cell surface markers.	25
Figure S2. Percentages of CD45+ and CD45- cells	26
Figure S3. Gating strategy for two subsets of granulocytes	27
Figure S4. Correlations between gestational age and major immune cell populations	28
Figure S5. Pairwise comparisons macrophage/monocyte cell clusters	30
Figure S6. Macrophage/monocyte abundances by infant sex	31
Figure S7. Correlations between gestational age and macrophage/monocyte clusters	33
Figure S8. Correlations between gestational age and T cell populations	34
Figure S9. T cell abundances by infant sex	35
Figure S10. Non-immune cell abundances by infant sex	36
Figure S11. Correlations between gestational age and non-immune cell clusters	37
Figure S12. PD-L1$^+$ non-immune cells by infant sex	38

Figure S13. PD-L1$^+$ non-immune cells by infant sex and gestational age in laboring and non-laboring pregnancies 40
References 41

Supplementary Methods

Panel Design and Antibody Titration. We designed a metal isotope antibody panel of 32 cell-surface markers, which could identify at least 22 canonical cell populations (Table S1). The panel was designed using MaxPar Panel Designer from Fluidigm Corporation. Metal isotope channels ranged from 209 to 75. Four antibodies in this panel, i.e. anti-HLA-G, -CD10, -Fas-L and -PD-L1(CD274), were conjugated with metal isotopes at the University of Chicago Cytometry and Antibody Technology Core Facility. The 32 antibodies were titrated in order to obtain the optimal amount of each antibody that maximally detected their targets on 3x10^6 decidual cells.

Protocol Modifications for Cell Preparation Before Antibody Staining. The variation in sample quality due to quality "sticky" samples clogging the machine resulted in poor data collection. These samples had an unusually large and variable amounts of cell debris and free DNA generated by tissue digestion, and mucus that can significantly impact the quality of single cell suspensions. To address these challenges, we developed a protocol for preparation and staining of human frozen decidual samples. Briefly, cells were thawed and then incubated with an accutase solution followed by DNase digestion. Cells were filtered and the debris removed using a density gradient (Debris Removal Solution from Milteny Biotec) prior to staining. Differences in data collection between a frozen decidual sample with and without treatments are shown in the figure below. Six million cells from each sample were split into two tubes (3 million cells each). Cells in tube 1 were prepared using the new protocol with DNase, accutase and cell debris removal treatment; cells in tube 2 were prepared following MaxPar® Cell Surface Staining Protocol (left panel). Cells in the tube 2 clogged the machine over time and negatively influenced signal detection and generating inaccurate frequencies of cell populations. For example, we obtained 16.9% of CD45$^+$ cells in the total cell population using our new protocol, whereas only 9.14% CD45$^+$ cells were collected following the conventional protocol (right panel). Finally, our new protocol significantly prevented clogging and improved overall run and data quality.

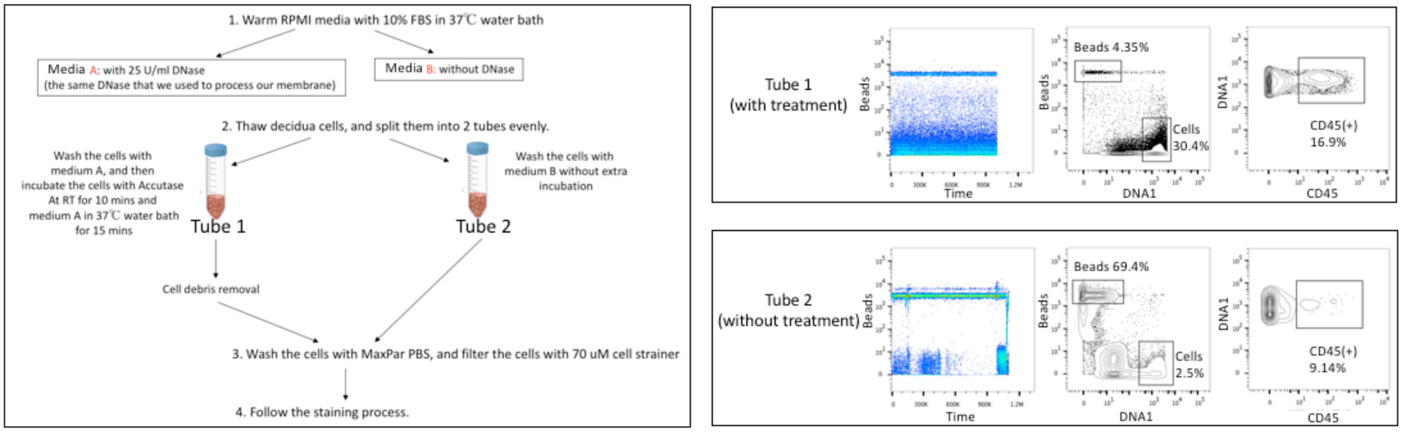

Protocol modification for "sticky" frozen decidual cells. (Left) Experimental workflow. Cells in Tube 1 were prepared following the new protocol with DNase, Accutase and cell debris removal treatment; cells in tube 2 were prepared following the traditional CyTOF staining protocol. (Right) Run quality control. Six representative scatterplots show comparisons between the two protocols.

Cell and Bead Staining. After washing by Maxpar Cell Staining Buffer twice, cells were incubated with Human TruStain FcX™ (BioLegend, Cat#422302) for 10 mins at room temperature. To avoid the competition of anti-CD3 and anti-γδ TCR staining, 1 ul γδ TCR antibody/sample was added and incubated with $3x10^6$ cells for 5 mins at room temperature. Then cells were stained with 100 ul of the antibody mix cocktail for 30 mins at room temperature and washed twice by Maxpar Cell Staining Buffer. Cells were fixed by 1.6% PFA/PBS (Thermo Fisher Scientific, Cat#28906) for 10 mins at room temperature and washed in Maxpar Cell Staining Buffer. Cell pellet was resuspended in 1 ml of Cell-ID™ Intercalator-Ir (Fluidigm, Cat#201192B) (4000x dilution of 500 µM stock solution) in Maxpar Fix & Perm Buffer (Fluidigm, Cat#201067) at 4ºC overnight. The next morning, cells were washed twice in Maxpar Cell Staining Buffer and twice in Maxpar water. Cell pellet was resuspended in Maxpar water with EQ™ Four Element Calibration Beads (Fluidigm, Cat#201078), and cell concentration was adjusted to 4.0x(10^5). Compensation beads were washed twice in Maxpar PBS and twice in Maxpar water and resuspended in 500 ul Maxpar water. Cells were filtered by 40 µM cell strainer (Fisher Scientific, Cat#07-201-430) before running on the mass cytometer.

Sample Quality Control. All samples included in these analyses went through a quality control check based on Fluidigm's QC guidance. In brief, the quality of Cell-ID Ir-Intercalator staining (DNA1 signal) was checked for each run. We required at least 50% of all events to be $DNA1^+$, to have enough beads (>1% of total events) to perform normalizations of the data, and the medium intensity of Eu153 on EQ4 beads to be greater than or equal to 1000. Samples that failed on any of these were discarded.

Testing of Signal Spillover. Although CyTOF technology does not produce fluorescence spillover between channels, metal signal spillover can occur due to impurities of antibodies, oxidation of metal isotopes, or machine sensitivity. To avoid false discoveries caused by spillover, we applied CATALYST (Table S7) and an interactive Shiny-based web application to test for signal spillover across all the channels for our panel. Briefly, polystyrene capture beads were single stained with each antibody used in the experiment. Beads were then pooled and analyzed simultaneously in the mass cytometer. Single stained controls for each dye were analyzed to determine the percentage of interfering signal in all the channels. These values are reported into a spillover matrix (see figure below).

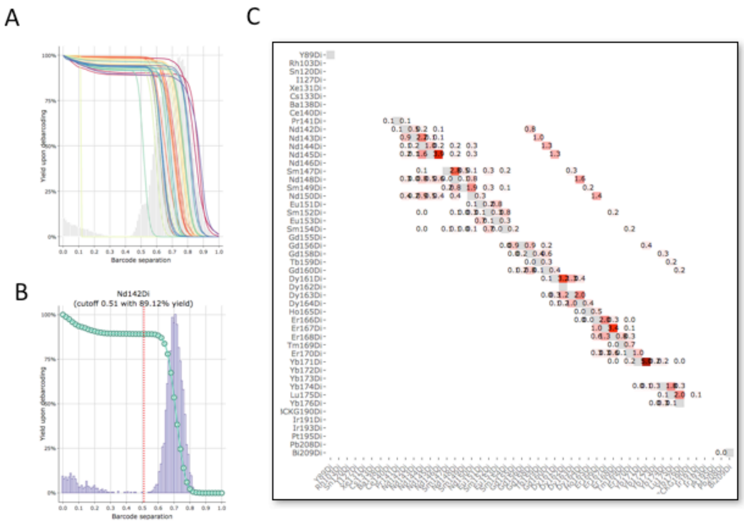

Compensating signal spillover. (A) The single bead-positive populations colored by metal isotopes in our 32 antibody panel were identified by default in CATALYST package. The bar graphs showed the distribution of cells corresponding to the barcode separation. (B) A representative (^{142}Nd chanel) example of the automatic estimation for each individual population. The dotted line shows a function of the applied separation cutoff corresponding to the yield upon debarcoding generated by CATALYST. (C) A signal spillover matrix was generated by staining of control antibody-capture beads, which was performed with one decidua sample in parallel. The matrix displayed the spillover in potential affected channels including M±1 and M±16. The numbers in the cells on the diagonales were 1, while others were the percentages of spillover by channels in X-axis into channels in Y-axis.

Testing for Batch Effects

Due to the variability of 'stickiness' between these cells, it was not feasible to pool multiple samples together and use the fluidigm barcoding/pooling system. Therefore, to assess batch effects and demonstrate consistency between runs of individual samples, we (1) we compared 4 aliquots of the same decidual cell sample run at 4 different times on the same day; (2) compared runs performed on 2 different days using 2 different aliquots (vials) of the same decidual cell sample. For these studies, one decidual sample from a term laboring pregnancy was split into 4 individual tubes and stained with the 32- antibody panel. Cells from each of the 4 tubes were sequentially run on the Helios machine over the course of one day. Analysis of $CD45^{+}$ and $CD45^{-}$ cell populations showed that all four samples performed similarly with respect to both cell frequency and signal intensity (see figure below, upper panel A). We also looked at the percentage of rare populations, such as γδ T cells and observed little variation between samples (see figure below, lower panel A). All four samples showed similar cellular distributions and clustering (see figure below, panel B). This experiment was repeated using a second sample and run on a different day, with similar results.

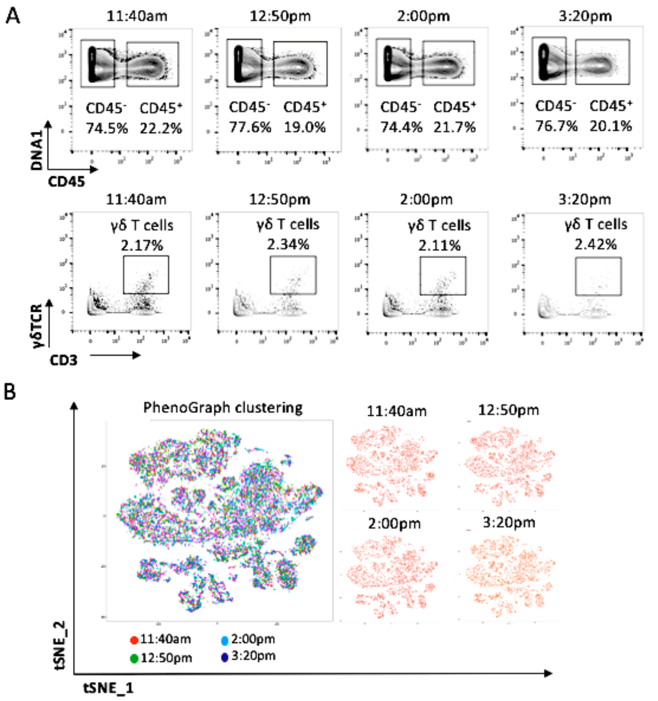

Test of batch effects between 4 samples of the same decidua run in one single day. (A) Comparison of the signal intensity and the percentage of live cells between runs of the same sample. Scatterplots on the first row represented the total live cells in one vial of decidual sample, which was evenly split into 4 tubes for antibody staining and CyTOF ran on the same day. Scatterplots on the second row shows the percentage of γδ T cells in population of CD45+ live decidua cells (B) The overlapped (colored) and individual (red) phenograph data visualization by cytofkit (R package), showing whole population structure among these 4 time points. Cellular distribution and clustering were defined by tSNE1 and tSNE2. 5000 viable single cells from each time point were subjected to PhenoGraph (B) in cytofkit.

Next, aliquots of cells from one term non-laboring sample were run on 2 different days. Again, the number of live cells and the percentage of CD45$^+$ cells between aliquots and runs were similar. Both runs yielded a similar cellular distribution and clustering (see figure below).

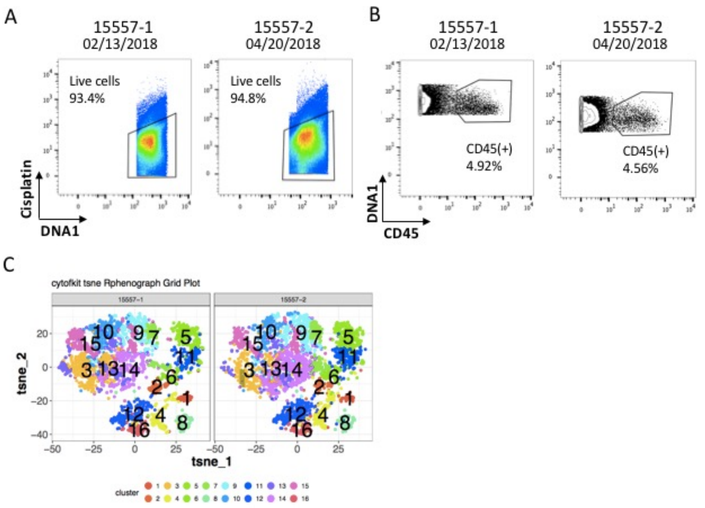

Test of batch effects between 2 aliquots of the same sample run on different days (2 months apart).
Signal intensity and percentage of total live cells (A) and CD45+ live cells (B) from one term non-laboring pregnancy. Scatterplots show the number of total live cells in each sample. (C) Rphenograph display of the distribution of the samples on each day by cytofkit (R package), defined by tSNE1 and tSNE2. 3000 viable cells from each day were subjected to PhenoGraph (C) in cytofkit.

Based on the results of these studies, we ran 4 samples a day for our study, one sample from each of the 4 biological groups (preterm and term laboring; preterm and term non-laboring). The order of the categories in each run were alternated to control for any remaining batch effects due to the order in which they are loaded into the machine.

CyTOF Data Analysis and Visualization. We primarily used Cytosplore (1) and Cytofkit (2, 3)

software to perform data analysis and data visualization (see Table S7).
Cytosplore (1): For Hierarchical Stochastic Neighbor Embedding (HSNE) analysis, live $CD45^{+}$/$CD45^{-}$ cells from each sample were gated, imported and analyzed on the Cytosplore platform using negative value pruned inverse hyperbolic sine transformation (cytofAsinh) transformation and HSNE visualization. The HSNE graphs and the marker expression heatmaps were exported for making figures. The matrix, including the frequency of each cell population, was exported as an excel file for statistical analysis.
Cytofkit (3): After installation of Cytofkit package and library, the command: “cytofkit_GUI()” was used to launch the GUI. The extracted .fcs files were imported and transformed using cytofAsinh in cytofkit. “Rphenograph” was chosen as the major cluster method, while “PCA” and “tSNE” were chosen as the visualization methods. After analysis, .RData files were saved and then loaded to Shiny APP for further exploring the data and results in an interactive manner. Launching the Shiny APP and loading .RDatwere followed usage of Shiny APP (Table S7).

Supplementary Results –

Gestational Age, Sex and Labor Effects on Cell Distributions

Immune Cell Lineages. In the combined sample of term and preterm pregnancies, the abundances of the major immune cell subsets did not differ significantly by gestational age (Supplementary Fig. 4A-B), but $CD3^+$ T cells (*P* = 0.004) and NK cells (*P* = 0.001) were more abundant in pregnancies with male infants and macrophages/monocytes (*P* = 0.040) were more abundant in pregnancies with female infants (Fig. 3A), consistent with earlier studies suggesting a more inflammatory or regulatory immune profile in pregnancies with male fetuses (4-7). The differences between male and female infants in our study may reflect the observed lower abundances of $CD3^+$ T cells (z= -2.47, *P* = 0.01) and NK cells (z= -2.45, *P* = 0.01) and a higher abundance of macrophages/monocytes (z= 2.57, *P* = 0.01) in TL (75% female) compared to TNL (55% female) and of NK cells in PNL (36% female) compared to PL (45% female) (z= 2.40, *P* = 0.02) (Fig. 3A; Supplementary Tables 3 and 5).

The abundance of granulocytes was higher in TL compared to TNL (z= 2.38, *P* = 0.02). None of the macrophage/monocyte cell clusters differed between pregnancies with male or female infants (Supplementary Fig. 6) or by gestational age (Supplementary Fig. 7).

T Cell Subsets. T cells in clusters 1 and 3, 9, 10 were overall more abundant in pregnancies with male infants. Because TL had fewer male pregnancies (25% male) compared to TNL (46% male), it is likely that the differences between TL and TNL for cells in clusters 1, 3, 9 and 10 reflected sex ratio imbalances and not true differences between laboring and non-laboring pregnancies at term. Clusters 2 and 7, defined as Th17 CD4 T cells and $HLA\text{-}DR^+PD\text{-}1^+$ CD8 T cells, were less abundant in TL compared to TNL (*P* = 0.0098 and $P = 7.43\times10^{-9}$, respectively; Fig. 5) and did not differ by either gestational age or infant sex in the combined sample. The same cells in cluster 7

were also higher in TNL compared to PNL (P = 0.0012), and in PL compared to TL, but these differences did not reach significance (P = 0.069) (Fig. 5; Supplementary Tables 3 and 5). Moreover, there was a non-signficant trend toward decreasing cell proportions with increasing gestation (p=0.28) in the laboring pregnancies, whereas the trend was opposite and approaching significance (p= 0.067) in the non-laboring group. However, the proportions of cluster 7 cells do not differ between the laboring and non-laboring groups at term (weeks 38-40), but cluster 7 cell proportions are much lower in the early preterm nonlaboring compared to early preterm laboring (weeks 25-30). Because the preterm non-laboring samples were all from women with severe preeclampsia, this observation suggests that these cell types may be unusually low in women with this condition or that these observations are due to random fluctuations in small samples.

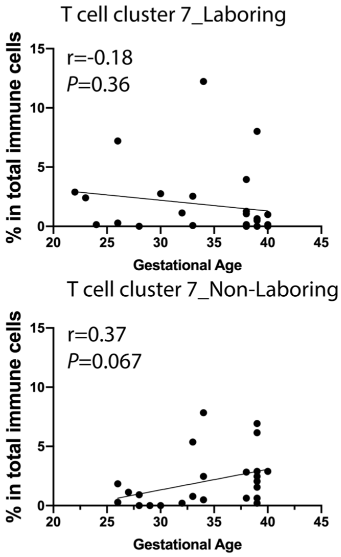

CD45- Non-Immune Cells. None of the non-immune cell clusters differed between pregnancies with male or female infants (Supplementary Fig. 10) or by gestational age (Supplementary Tables , although cells in unidentified cluster 1 trended toward decreasing abundance with increasing gestational age (r = 0.25; P = 0.08) (Supplementary Fig. 11 and Supplementary Tables 4-5).

PD-L1⁺ Non-Immune Cells. The abundances of PD-L1⁺ non-immune cells increased with increasing gestational age among laboring pregnancies (r=0.037, P = 0.057 (Supplementary Fig. 13A),

but did not change in non-laboring pregnancies (r = 0.13*,* *P* = 0.53 (Supplementary Fig. 13B). The latter observation suggests that the abundances of PD-L1^{+} non-immune cells at the MFI are not influenced by gestational age per se but rather may be a feature of labor at term. Moreover, infant sex was not correlated with gestational age at delivery in either the laboring (male infants: r = 0.41; *P* = 0.23; female infants: r = 0.35; *P* = 0.16) or non-laboring (male infant: r = 0.13; *P* = 0.65; female infant: r = 0.31; *P* = 0.35) pregnancies (Supplementary Fig. 13C-D). Thus, fetal sex was not likely contributing to the differences in abundances of PD-LI^{+} non-immune cells between TL and PL (Fig. 6C).

Supplementary Tables

Table S1. CyTOF panel of 32 cell-surface markers for decidual tissues. MCs: monocytes, DCs: dendritic cells, mDCs: myeloid dendritic cells, pDCs: plasmacytoid dendritic cells, EVTs: extravillous trophoblasts.

Antigen	Clone	Tag	Source	Identifier	Target
CD33	WM53	169Tm	Fluidigm	Cat#3169010B	Cells of myeloid lineage
CD14	M5E2	160Gd	Fluidigm	Cat#3160001B	Classic MCs
CD45RA	HI100	143Nd	Fluidigm	Cat#3143006B	Naïve T cells
CD25/lL-2R	2A3	149Sm	Fluidigm	Cat#3149010B	Activated and proliferated T cells
CD8a	SK1	168Er	Fluidigm	Cat#3168002B	CD8 T cells
CD11b/Mac-1	ICRF44	209Bi	Fluidigm	Cat#3209003B	Macrophages, DCs and eosinophils
TCRγδ	11F2	152Sm	Fluidigm	Cat#3152008B	γδ T cells
HLA-DR	L243	174Yb	Fluidigm	Cat#3174001B	Activated T cells, macrophages and DCs
CD56/NCAM	NCAM16.2	176Yb	Fluidigm	Cat#3176001B	NK cells and NK T cells
CD19	HIB19	142Nd	Fluidigm	Cat#3142001B	Immature and mature B cells
CD4	RPA-T4	145Nd	Fluidigm	Cat#3145001B	CD4 T cells
CD11c	Bu15	147Sm	Fluidigm	Cat#3147008B	mDCs and neutrophils
CD16	3G8	148Nd	Fluidigm	Cat#3148004B	Intermediate and non-classic MCs, macrophages
CD123 /lL-3R	6H6	151Eu	Fluidigm	Cat#3151001B	pDCs
CD45	HI30	89Y	Fluidigm	Cat#3089003B	Leukocytes
CD3	UCHT1	170Er	Fluidigm	Cat#3170001B	Pan T cells
CD66a/CEACAM 1	CD66a-B1.1	171Yb	Fluidigm	Cat#3171004B	Granulocytes
CD7	M-T701	166Er	Fluidigm	Cat#3166027B	NK cells and T cells
CD69	FN50	144Nd	Fluidigm	Cat#3144018B	Tissue-resident/early activated T cells
CD196/CCR6	G034E3	141Pr	Fluidigm	Cat#3141003A	Th17 and Th22 $CD4^+$ T cells
CD195/CCR5	NP-6G4	156Gd	Fluidigm	Cat#3156015A	Th1 $CD4^+$ T cells
CD183/CXCR3	G025H7	163Dy	Fluidigm	Cat#3163004B	Th1 $CD4^+$ T cells, eosinophils and other cells
CD194/CCR4	205410	153Eu	Fluidigm	Cat#3153013A	Th2, Th17 and Th22 $CD4^+$ T cells
NK1.1/CD161	HP-3G10	159Tb	Fluidigm	Cat#3159004B	NK cells, NK T cells and IL-17 producing cells
CD279/PD-1	EH12.2H7	175Lu	Fluidigm	Cat#3175008B	Exhausted T cells
CD95/Fas	DX2	164Dy	Fluidigm	Cat#3164008B	Apoptotic cells
CD169	7-239	158Gd	Fluidigm	Cat#3158027B	Tissue-resident macrophages
CD45RO	UCHL1	165Ho	Fluidigm	Cat#3165011B	Memory T cells
CD178/FasL	NOK1	154Sm	Bio-Legend	Cat#306409	Stromal cells, CTBs and cytotoxic T cells
HLA-G	87G	167Er	Bio-Legend	Cat#335904	CTBs
CD10	HI10a	161Dy	Bio-Legend	Cat#312223	Stromal cells and CTBs
CD274/PD-L1	29E.2A3	150Nd	Bio-Legend	Cat#329719	Stromal cells, CTBs, and suppressive T cells and macrophages

Table S2. Cell-surface markers for identifying immune and non-immune lineages in 2-level HSNE analysis.

Filters	Markers
1st Filter: CD45+	CD11c, CD123, CD169, CD14, CD7, CD16, CD33, CD3, CD66a, HLA-DR, CD56, CD161/NK1.1, CD11b, CD19
1st Filter: CD45-	CD10, HLA-G, CCR4, CCR5, CCR6, CXCR3, PD-L1, Fas-L, CD66a, PD-1, HLA-DR
2nd Filter: T cells	CD8, CD4, CD69, CCR4, CCR5, CCR6, CXCR3, PD-1, HLA-G, HLA-DR, PD-L1, Fas-L, CD161/NK1.1, CD11c, CD25, γδTCR, CD45RA, CD45RO
2nd Filter: Macrophages/Monocytes	CD16, CD14, PD-L1, CD4, CCR4, CCR5, CCR6, CXCR3, CD169, HLA-DR, CD11b
2nd Filter: Granulocyte	CD66a, CD11B, CD11c, CD16, CXCR3

Table S3. Immune cell pairwise comparisons. Z-scores and p-values from Robust Rank-Order tests. *P*-values <0.06 are shown in bold font.

**A. Major T cell clusters**								
	**TL vs PL**		**TL vs TNL**		**PL vs PNL**		**TNL vs PNL**	
	**z**	**p-value**	**z**	**p-value**	**z**	**p-value**	**z**	**p-value**
Pan T cells	-1.56	0.12	-2.47	**0.014**	0.31	0.76	0.52	0.60
CD4 T cells	0.55	0.58	-1.12	0.26	-1.39	0.16	0.27	0.79
CD8 T cells	-1.48	0.14	-2.05	**0.041**	0.43	0.67	1.01	0.31
Macrophages/Monocytes	0.38	0.70	2.57	**0.010**	1.19	0.23	-0.70	0.49
mDCs	-0.29	0.77	1.14	0.25	0.33	0.74	-0.78	0.44
NK cells	-0.89	0.37	-2.45	**0.014**	-2.40	**0.017**	0.42	0.67
Granulocytes	0.17	0.86	2.38	**0.017**	0.93	0.35	-0.35	0.72
B cells	1.16	0.25	0.86	0.39	-0.92	0.36	-0.53	0.60
**B. T cell clusters**								
	**z**	**p-value**	**z**	**p-value**	**z**	**p-value**	**z**	**p-value**
cluster 1	-1.54	0.12	-2.86	**0.0042**	0.72	0.47	1.82	0.069
cluster 2	-0.50	0.62	-2.58	**0.0098**	0.14	0.89	1.24	0.21
cluster 3	-1.89	**0.059**	-2.04	**0.042**	0.43	0.67	0.75	0.45
cluster 4	0.28	0.78	-0.72	0.47	-0.57	0.57	0.078	0.94
cluster 5	-0.13	0.39	-1.45	0.15	-0.62	0.53	0.49	0.62
cluster 6	-1.20	0.23	-1.84	0.066	0.36	0.72	0.83	0.40
cluster 7	-1.82	0.069	-5.78	**7.43×10^{-9}**	0.91	0.36	2.52	**0.012**
cluster 8	0.88	0.38	0.53	0.60	-0.11	0.91	0.14	0.89
cluster 9	-1.49	0.14	-2.10	**0.036**	-0.13	0.89	0.24	0.81
cluster 10	-1.35	0.18	-3.21	**0.0013**	-0.32	0.75	0.057	0.95
**C. Macrophage/Monocyte clusters**								
	**z**	**p-value**	**z**	**p-value**	**z**	**p-value**	**z**	**p-value**
cluster 1	-0.97	0.33	-1.25	0.21	-0.19	0.85	0.056	0.96
cluster 2	0.38	0.70	0.82	0.41	2.64	**0.0084**	3.03	**0.0025**
cluster 3	0.15	0.88	1.11	0.27	0.17	0.86	-0.75	0.45
cluster 4	1.72	0.085	0.49	0.63	0.14	0.89	0.94	0.35
cluster 5	0.27	0.79	2.02	**0.043**	0.36	0.72	-0.34	0.74

Table S4. **Non-immune cell pairwise comparisons.** Z-scores and p-values from Robust Rank-Order tests. *P*-values <0.06 are shown in bold font.

**A.** Non-immune cells								
	**TL vs PL**		**TL vs TNL**		**PL vs PNL**		**TNL vs PNL**	
	**z**	**p-value**	**z**	**p-value**	**z**	**p-value**	**z**	**p-value**
DSCs cluster 1	-0.83	0.41	-0.96	0.34	-0.74	0.46	0.21	0.83
DSCs cluster 2	0.12	0.90	0.19	0.85	0.85	0.40	0.29	0.77
EVTs cluster 1	2.03	**0.042**	0.76	0.45	0.057	0.95	0.64	0.52
EVTs cluster 2	0.36	0.72	-1.43	0.15	-1.62	0.11	0.44	0.66
Undefined cluster 1	0.15	0.88	1.20	0.23	0.45	0.65	-0.79	0.43
Undefined cluster 2	-1.94	**0.053**	-0.23	0.81	0.54	0.59	-0.45	0.65
**B.** PD-L1 non-immune cells								
	**z**	**p-value**	**z**	**p-value**	**z**	**p-value**	**z**	**p-value**
PD-L1+ non-immune cells	2.70	**0.0070**	0.26	0.80	0.083	0.93	0.82	0.41

Table S5. Distributional characteristics of cell clusters.

	Term Labor (N=16)			Preterm Labor (N=11)			Term Non-Labor (N=11)			Preterm Non-Labor (N=14)		
**Immune Cells**	Median	25% Percentile	75% Percentile	Median	25% Percentile	75% Percentile	Median	25% Percentile	75% Percentile	Median	25% Percentile	75% Percentile
pan T cells	14.85	5.34	27.85	23.60	19.80	48.10	36.30	19.40	48.40	23.35	12.10	42.80
Macrophages/ Monocytes	50.15	28.25	76.05	46.60	18.10	61.50	25.40	14.58	39.30	24.90	14.90	50.38
NK cells	2.60	1.15	8.60	5.70	2.80	13.70	18.30	2.90	21.50	11.05	5.98	20.00
mDCs	2.58	1.23	4.85	3.10	1.10	5.00	2.20	1.10	3.40	2.95	1.55	8.10
Granulocytes	4.04	2.38	9.48	2.60	1.00	9.80	1.70	1.14	3.60	1.80	1.08	6.10
B cells	0.15	0.00	0.60	0.00	0.00	0.20	0.00	0.00	0.38	0.05	0.00	0.55
**Macrophages/ Monocytes**												
cluster 1	0.31	0.14	1.51	0.78	0.26	4.91	0.99	0.29	4.54	1.36	0.12	3.24
cluster 2	1.17	0.16	6.29	1.33	0.51	2.50	0.68	0.27	2.23	0.19	0.05	0.85
cluster 3	0.99	0.40	4.57	1.30	0.56	3.34	0.63	0.38	2.16	0.68	0.25	3.76
cluster 4	0.26	0.14	7.26	0.11	0.04	0.34	0.23	0.08	0.36	0.24	0.03	2.30
cluster 5	0.84	0.39	1.70	0.84	0.10	3.72	0.39	0.13	0.87	0.32	0.09	1.50
**T cells**												
Cluster 1	0.83	0.45	1.55	1.76	1.14	2.61	2.04	1.08	3.80	1.28	0.64	2.67
Cluster 2	0.33	0.00	1.61	1.51	0.00	2.77	2.77	0.63	4.28	0.57	0.00	4.06
Cluster 3	0.70	0.17	1.41	1.91	0.33	2.65	2.65	0.78	3.31	0.83	0.23	2.20
Cluster 4	1.33	0.39	2.44	1.29	1.04	2.37	1.50	0.87	2.91	1.54	0.69	3.63
Cluster 5	0.70	0.17	1.41	1.41	0.66	1.95	2.12	0.96	3.53	1.00	0.43	3.28
Cluster 6	0.61	0.16	1.12	1.76	0.00	3.69	2.89	0.32	4.81	0.96	0.44	2.12
Cluster 7	0.14	0.00	1.04	2.40	0.14	2.90	2.47	0.64	2.90	0.78	0.16	2.00
Cluster 8	2.05	0.68	3.57	1.49	0.87	2.11	1.74	0.87	2.73	1.62	1.06	2.05
Cluster 9	0.82	0.35	1.34	1.58	0.69	2.72	1.64	1.14	2.21	1.36	0.85	2.68
Cluster 10	0.55	0.22	1.35	1.45	0.07	2.28	2.28	0.76	2.49	1.21	0.07	3.75
**Non-immune Cells**												
DSC cluster 1	12.60	8.15	22.10	23.50	7.10	26.50	22.10	9.00	43.10	22.10	8.18	37.03
**DSC cluster 2**	9.80	3.65	15.55	6.00	2.40	16.50	4.70	3.60	17.00	5.45	3.50	8.05
**EVT cluster 1**	27.40	18.40	54.18	21.60	10.90	28.60	17.10	13.70	50.50	17.25	7.60	45.10
**EVT cluster 2**	1.30	0.30	3.00	1.70	0.30	2.10	2.90	0.80	7.30	2.15	0.48	7.93
Undefined cluster 1	22.35	11.10	34.73	21.20	18.20	35.00	15.80	13.70	24.80	17.65	11.33	36.38
Undefined cluster 2	10.90	7.20	15.20	14.10	12.20	19.00	12.00	8.90	14.40	12.85	7.60	19.00
**PDL1+ non-immune cells**												
	36.5	30.38	58.23	25.3	19	35.1	44.5	20	60.3	21.4	30.38	53.28

Table S6. T cell and NK cell abundances by labor type (A) and term/preterm (B)

A. Labor vs. Non-Labor

T cells

	Labor	Non-Labor
Males	31.65	24.75
Females	12.10	22.30
**Male/Female**	**2.62**	**1.11**

NK cells

	Labor	Non-Labor
Males	7.27	18.95
Females	2.14	6.30
**Male/Female**	**3.40**	**3.01**

B. Term vs. Preterm

T cells

	Preterm	Term
Males	25.20	37.30
Females	18.30	12.70
**Male/Female**	**1.38**	**2.94**

NK cells

	Preterm	Term
Males	7.30	9.70
Females	5.65	2.40
**Male/Female**	**1.29**	**4.04**

Table S7. Phenotype keys for 10 T-cell clusters.

Clusters	T cell subtype	Phenotype
1	Th2 CD4 T cells	$CXCR4^{high}CD4^{+}CD69^{+}CD8^{-}CD3^{+}$
2	Th17 CD4 T cells	$CXCR4^{high}CD161^{+}CD4^{+}CD69^{+}CD8^{-}CD3^{+}$
3	$CD11c^{+}HLA\text{-}G^{+}CD25^{low}$ CD8 T cells	$CD11c^{+}HLA\text{-}G^{+}CD25^{low}CD8^{+}CD69^{+}CD4^{-}CD3^{+}$
4	$CCR4^{+}CXCR3^{+}$ CD8 T cells	$CCR4^{+}CXCR3^{+}CD8^{+}CD69^{+}CD4^{-}CD3^{+}$
5	$\gamma\delta$ T cells	$TCR\gamma\delta^{+}CD8^{-}CD69^{+}CD4^{-}CD11c^{low}CD3^{+}$
6	$CD25^{+}HLA\text{-}DR^{+}$ CD4 T cells	$CD25^{+}HLA\text{-}DR^{+}PD\text{-}1^{+}CD45RO^{+}CCR4^{+}CCR5^{+}CD4^{+}CD69^{+}CD8^{-}CD3^{+}$
7	$HLA\text{-}DR^{+}PD\text{-}1^{+}$ CD8 T cells	$HLA\text{-}DR^{+}PD\text{-}1^{+}CCR5^{+}CD8^{+}CD69^{low}CD4^{-}CD3^{+}$
8	$CXCR3^{+}CCR5^{low}$ CD8 T cells	$CXCR3^{+}CCR4^{+}CD8^{+}CD69^{+}CD4\text{-}CD3^{+}$
9	$CD45RA^{-}CCR5^{low}$ CD8 T cells	$CD43RA^{-}CCR5^{low}CD8^{+}CD69^{+}CD4^{-}CD3^{+}$
10	Th1 CD4 T cells	$CCR5^{high}CD4^{+}CD69^{+}CD8^{-}CD3^{+}$

Table S8. Phenotype keys for 5 macrophage/monocyte clusters.

Clusters	Macrophage/monocyte cell sub-type	Phenotype
1	Classical monocytes	$CD14+CD16^{low}/CD169^{-}HLA\text{-}DR^{+}CD11b^{+}$
2	$PD\text{-}L1^{+}CXCR3^{high}CCR4^{high}$ tissue-resident macrophages	$CXCR4^{high}CXCR3^{high}CD169^{+}PD\text{-}L1^{+}CCR5^{low}HLA\text{-}DR^{+}CD11b^{+}$
3	$PD\text{-}L1^{-}CXCR3^{high}CCR4^{high}$ tissue-resident macrophages	$CXCR4^{high}CXCR3^{high}CD169^{+}PD\text{-}L1^{-}CCR5^{low}HLA\text{-}DR^{+}CD11b^{+}$
4	$CD4^{+}$ tissue-resident macrophages	$CD4^{+}CXCR4^{+}CXCR3^{+}CD169^{+}PD\text{-}L1^{-}CCR5^{low}HLA\text{-}DR^{+}CD11b^{+}$
5	$PD\text{-}L1^{-}CXCR3^{low}CCR4^{low}$ tissue-resident macrophages	$CXCR4^{low}CXCR3^{low}CD169^{+}PDL1^{+}CCR5^{low}HLA\text{-}DR^{+}CD11b^{+}$

Table S9. Software and algorithms.

Software/Algorithms	Website
**Normalizer v0.3**	https://github.com/nolanlab/bead-normalization/releases
**CATALYST**	https://bioconductor.org/packages/cytofkit/
**MATLAB Compiler Runtime (MCR) Release R2013b (8.2)**	https://www.mathworks.com/products/compiler/matlab-runtime.html
**R 3.5.1**	https://www.r-project.org/
**Cytofkit**	https://bioconductor.org/packages/cytofkit/
**Cytosplore**	https://www.cytosplore.org/
**FlowJo vX.07**	https://www.flowjo.com/
**GraphPad Prism 7**	https://www.graphpad.com/scientific-software/prism/

Supplementary Figures

Figures S1. H-SNE maps of cell surface markers. (A) H-SNE map was labeled by expression of 14 immune cell surface markers for annotation for the CD45+ immune compartment. (B) H-SNE map was labeled by expression of 11 cell surface markers for annotation for the CD45- non-immune compartment.

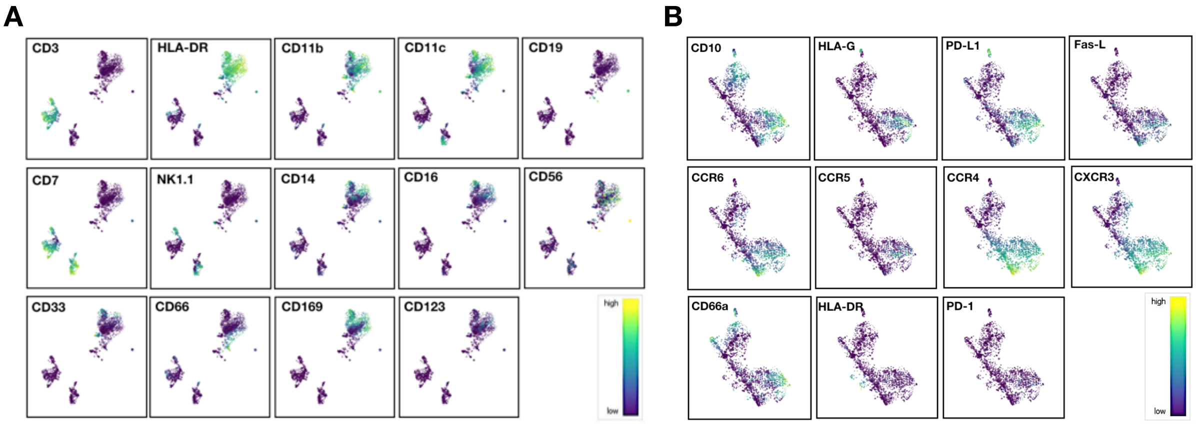

Figure S2. Percentages of CD45+ and CD45- cells in the four comparison groups. Median percentage of CD45+ cells in the four groups were 12.50, 12.38, 18.53, and 11.41, respectively; median percentage of CD45- cells in the four groups were 86.63, 87.25, 18.81, and 87.56, respectively.

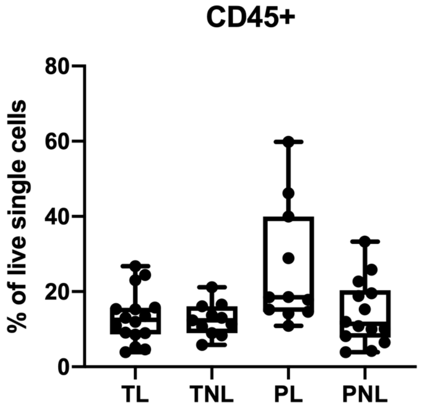

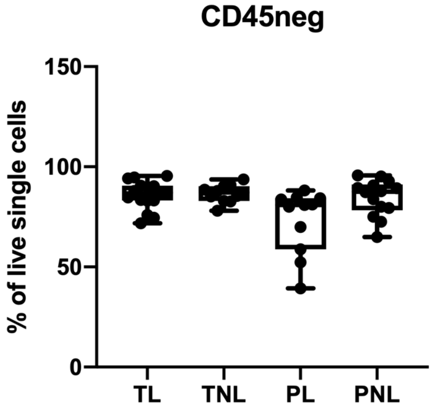

Figures S3. Gating strategy for two subsets of granulocytes.

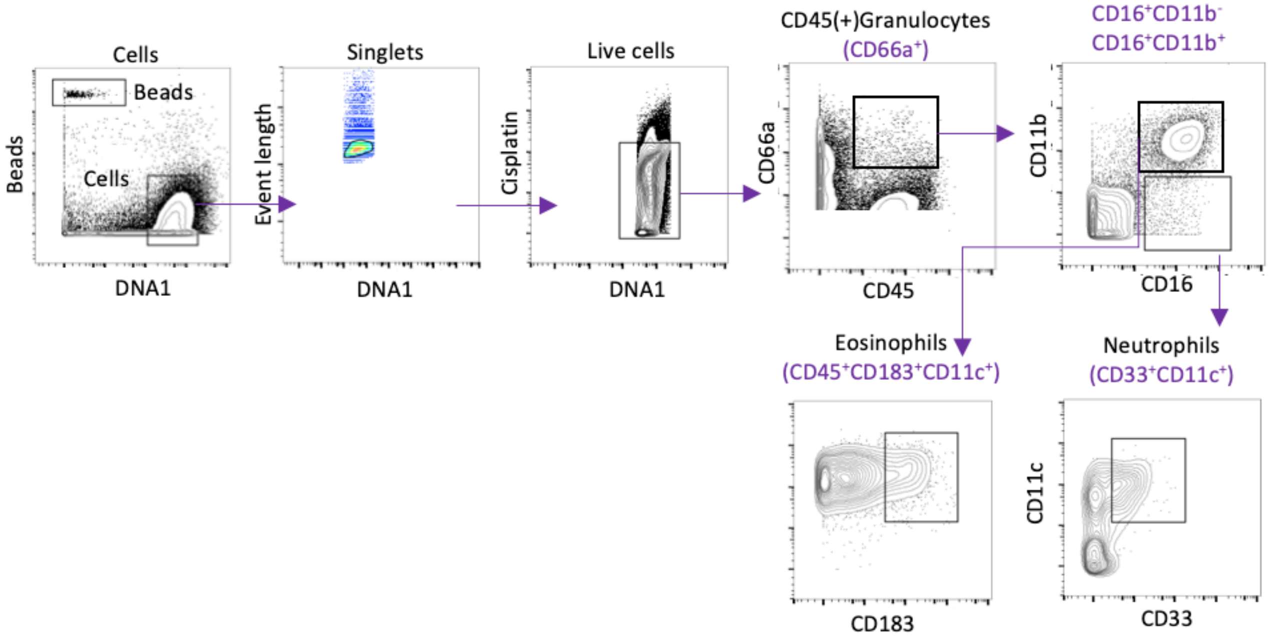

Figures S4. Correlations major immune cell populations by gestational age (A) and by comparison groups (B).

A)

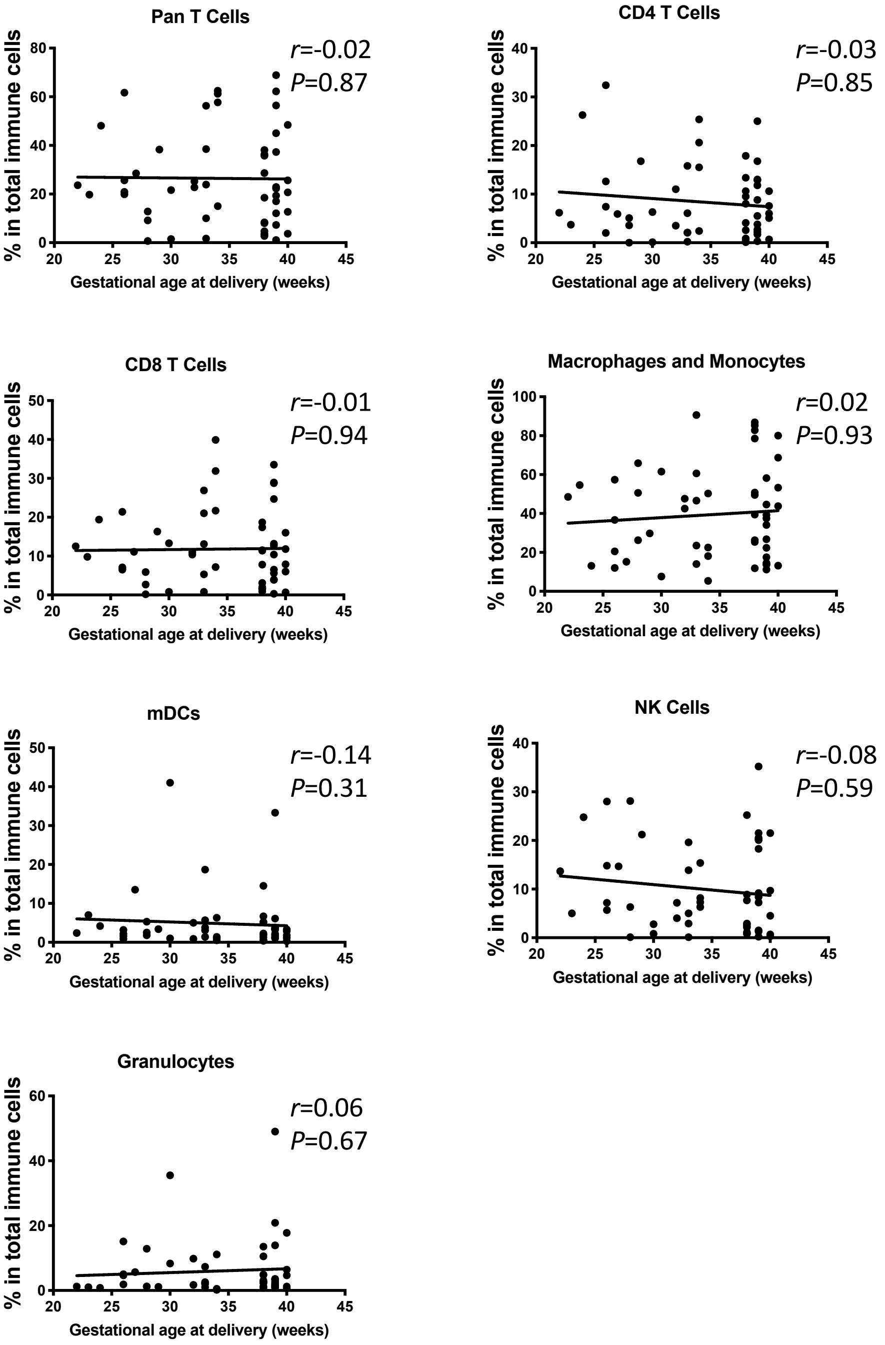

B)

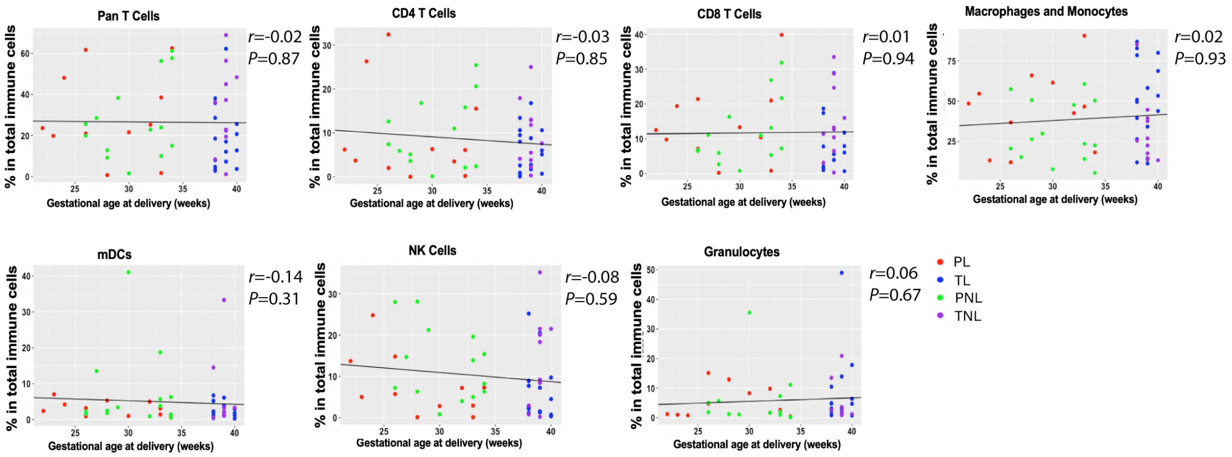
Pan T Cells
r=-0.02
P=0.87
CD4 T Cells
r=-0.03
P=0.85
CD8 T Cells
r=0.01
P=0.94
Macrophages and Monocytes
r=0.02
P=0.93
mDCs
r=-0.14
P=0.31
NK Cells
r=-0.08
P=0.59
Granulocytes
r=0.06
P=0.67
% in total immune cells
Gestational age at delivery (weeks)
PL
TL
PNL
TNL

Figures S5. Cell abundances by the four comparison groups for macrophage/monocyte cell clusters (A) and by gestational age. *P*-values <0.05 are shown.

A)

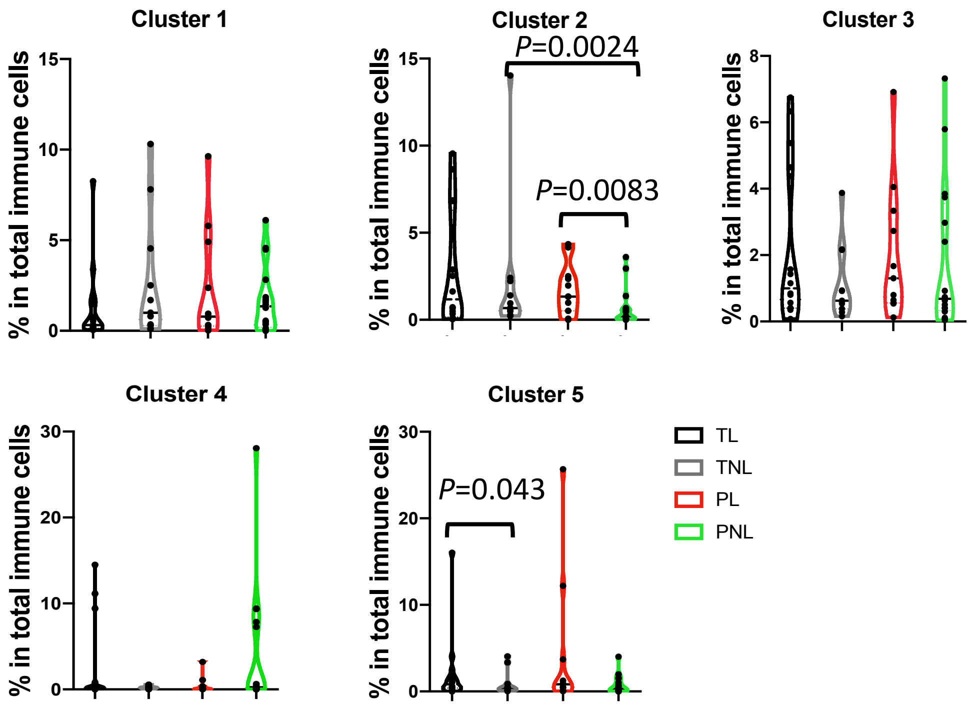

B)

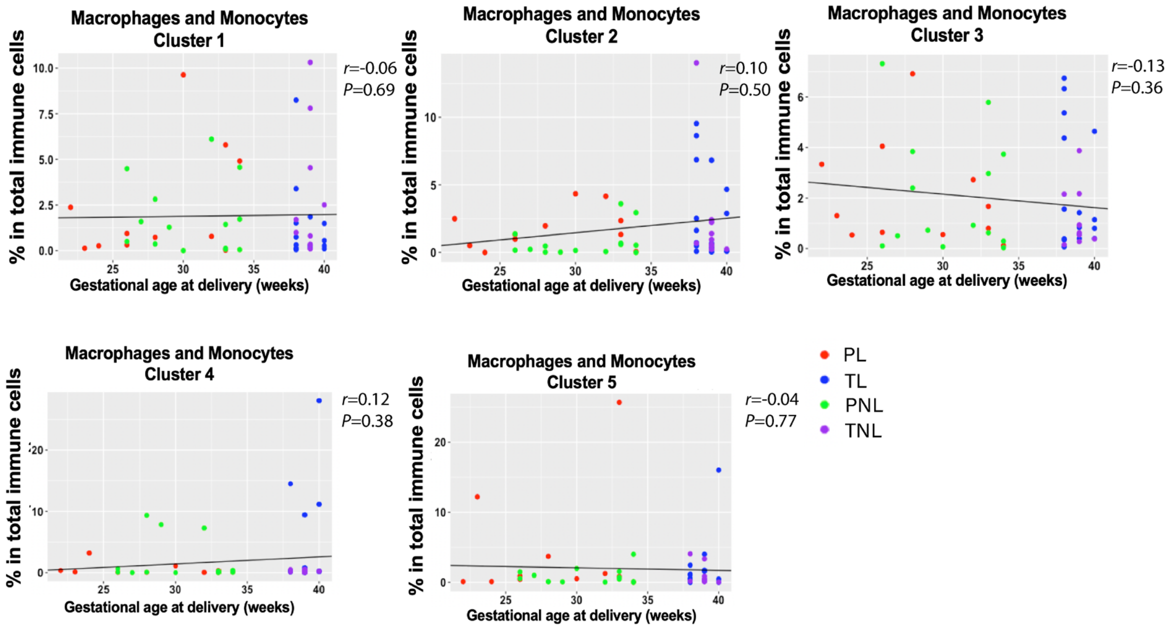

Figures S6. Macrophage/monocyte cell cluster abundances by infant sex (A) and by the four comparison groups (B).

A)

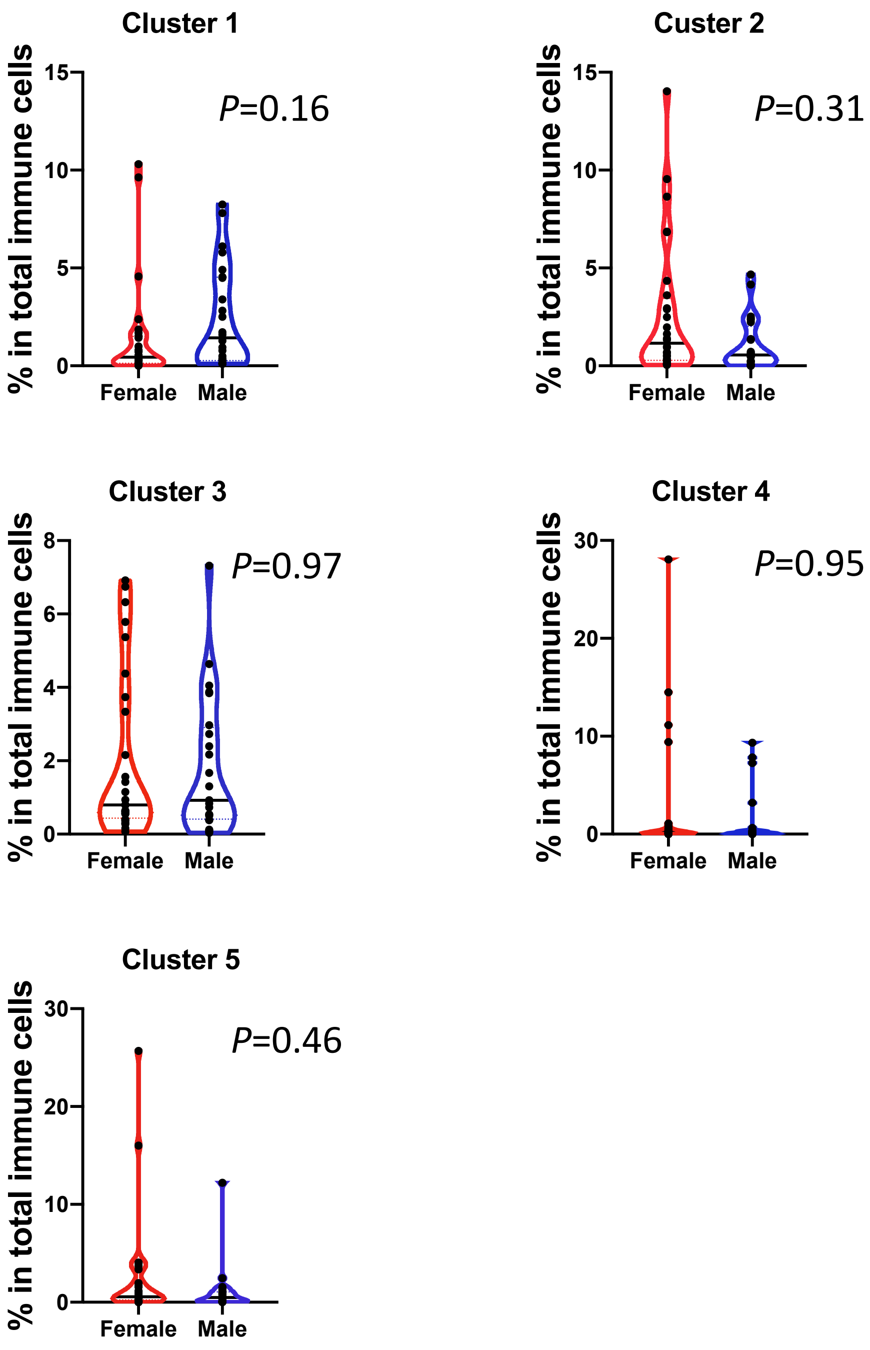

B)

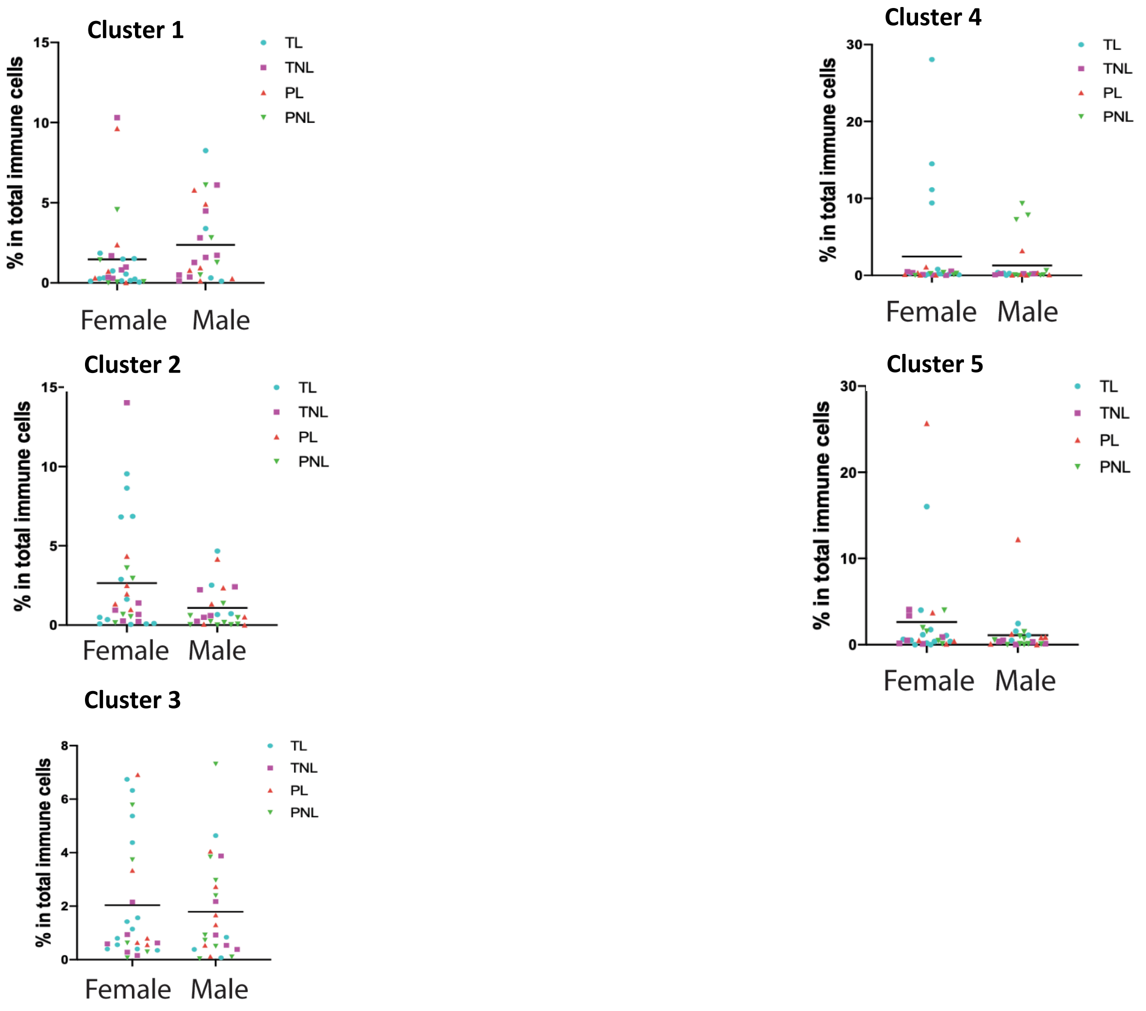

Cluster 1
Cluster 2
Cluster 3
Cluster 4
Cluster 5
% in total immune cells
Female
Male
TL
TNL
PL
PNL
0
5
10
15
0
2
4
6
8
0
10
20
30

Figures S7. Correlations between gestational age and macrophage/monocyte clusters.

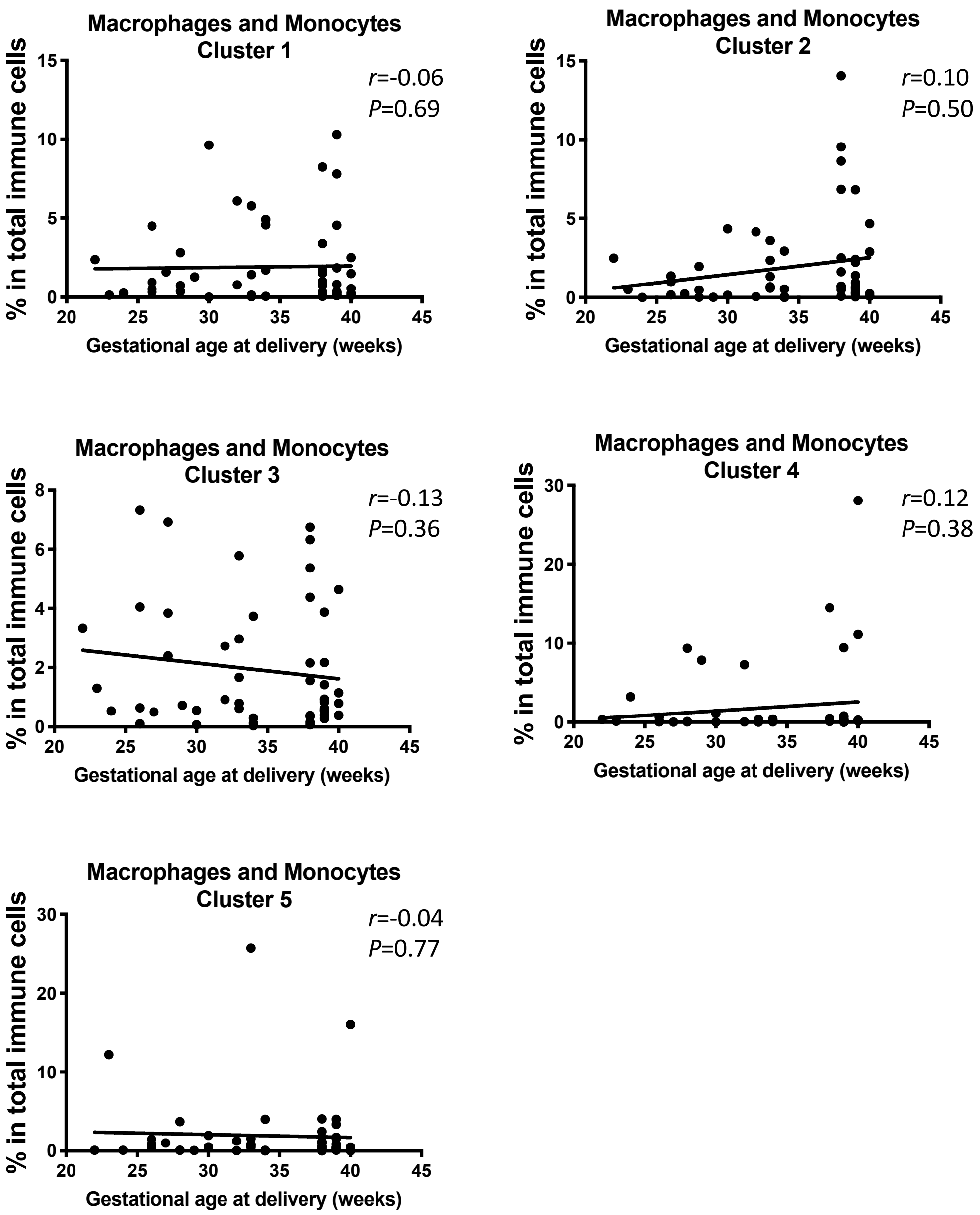

Figures S8. Correlations between gestational age and T cell populations by study group.

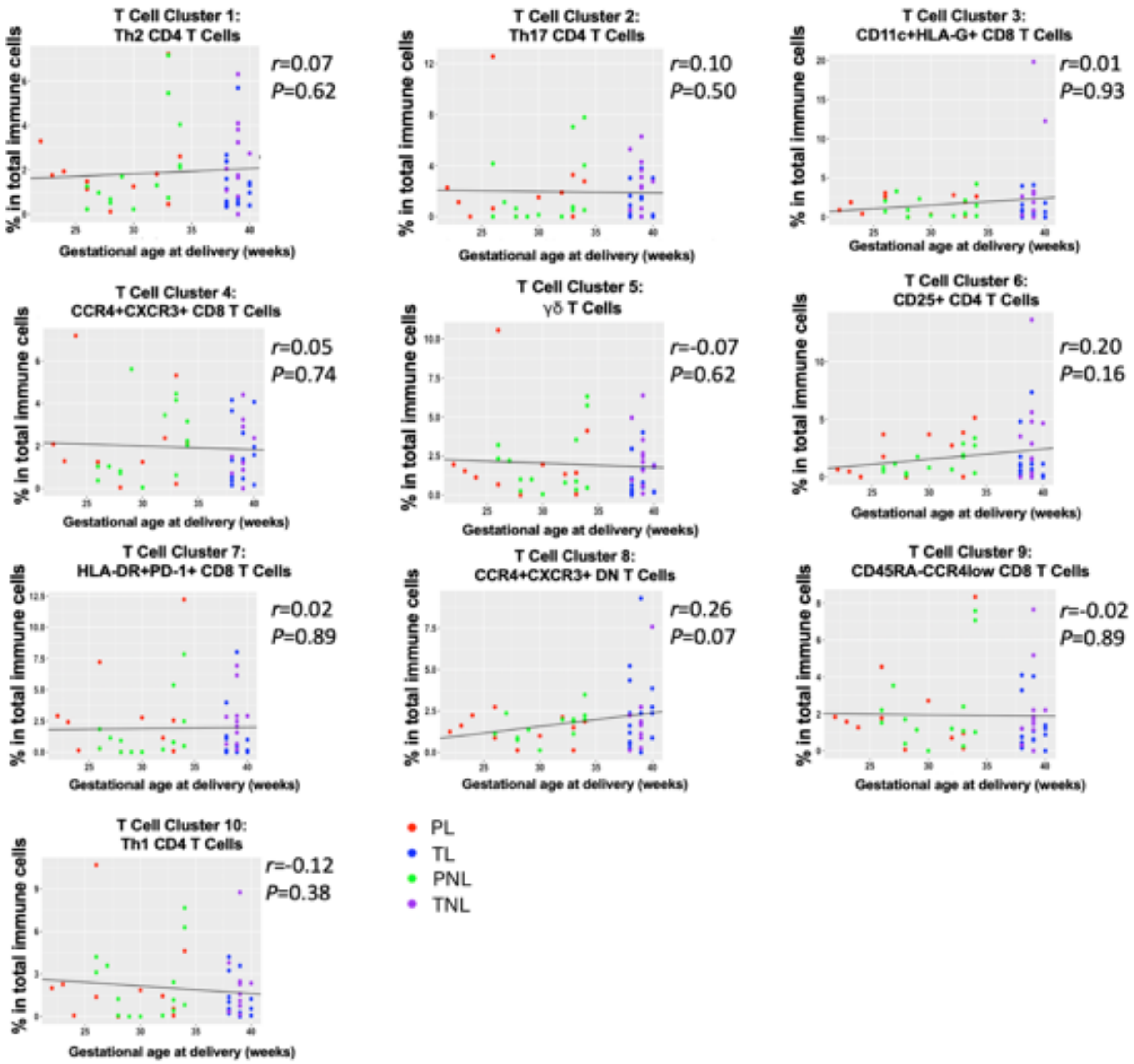

Figures S9. T cell abundances by infant sex and by the four comparison groups. *P*-values <0.05 are shown in bolded font (from Robust Rank-Order test).

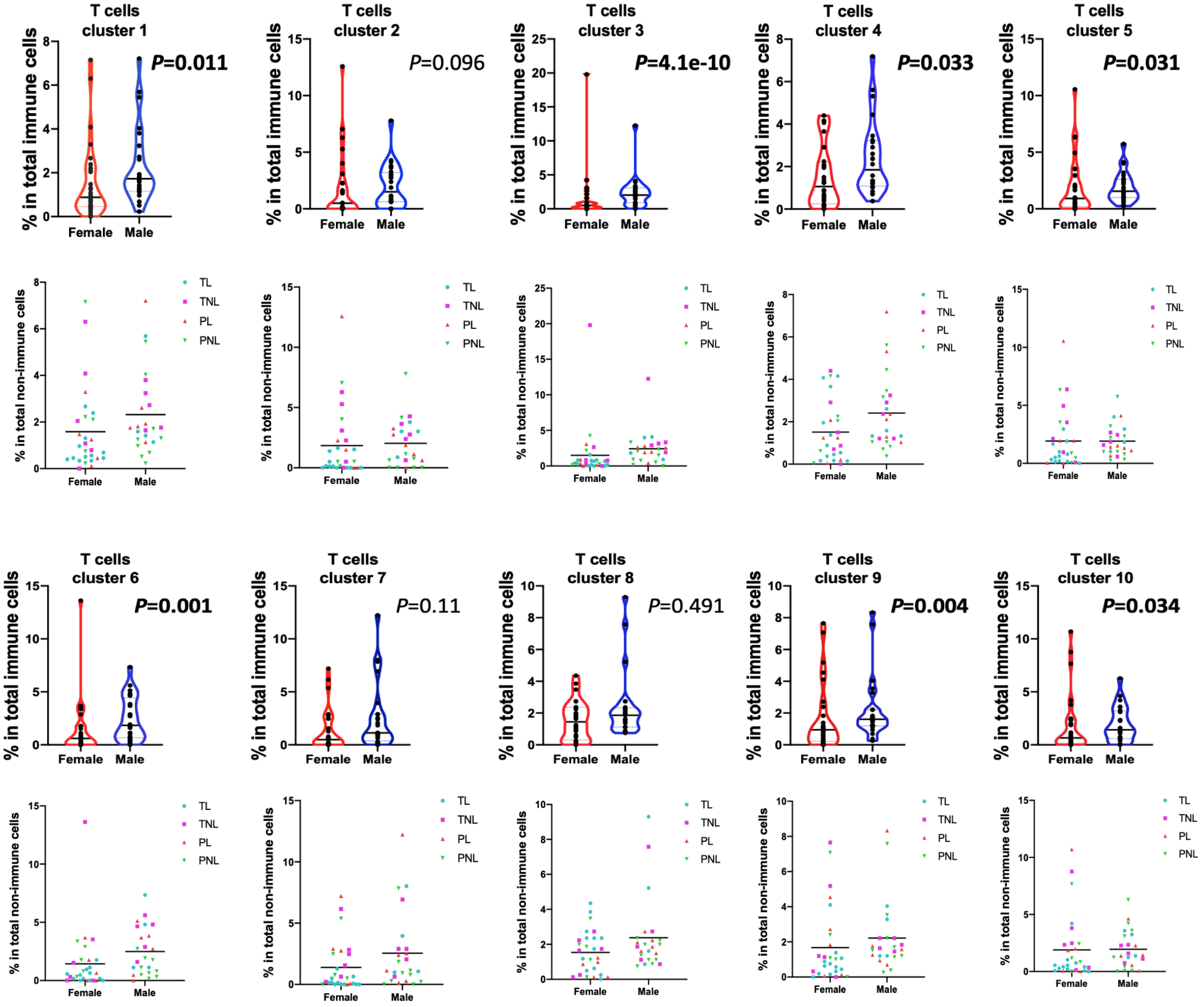

Figures S10. Non-immune cell abundances by infant sex and by the four comparison groups (B). *P*-values from Robust Rank-Order test.

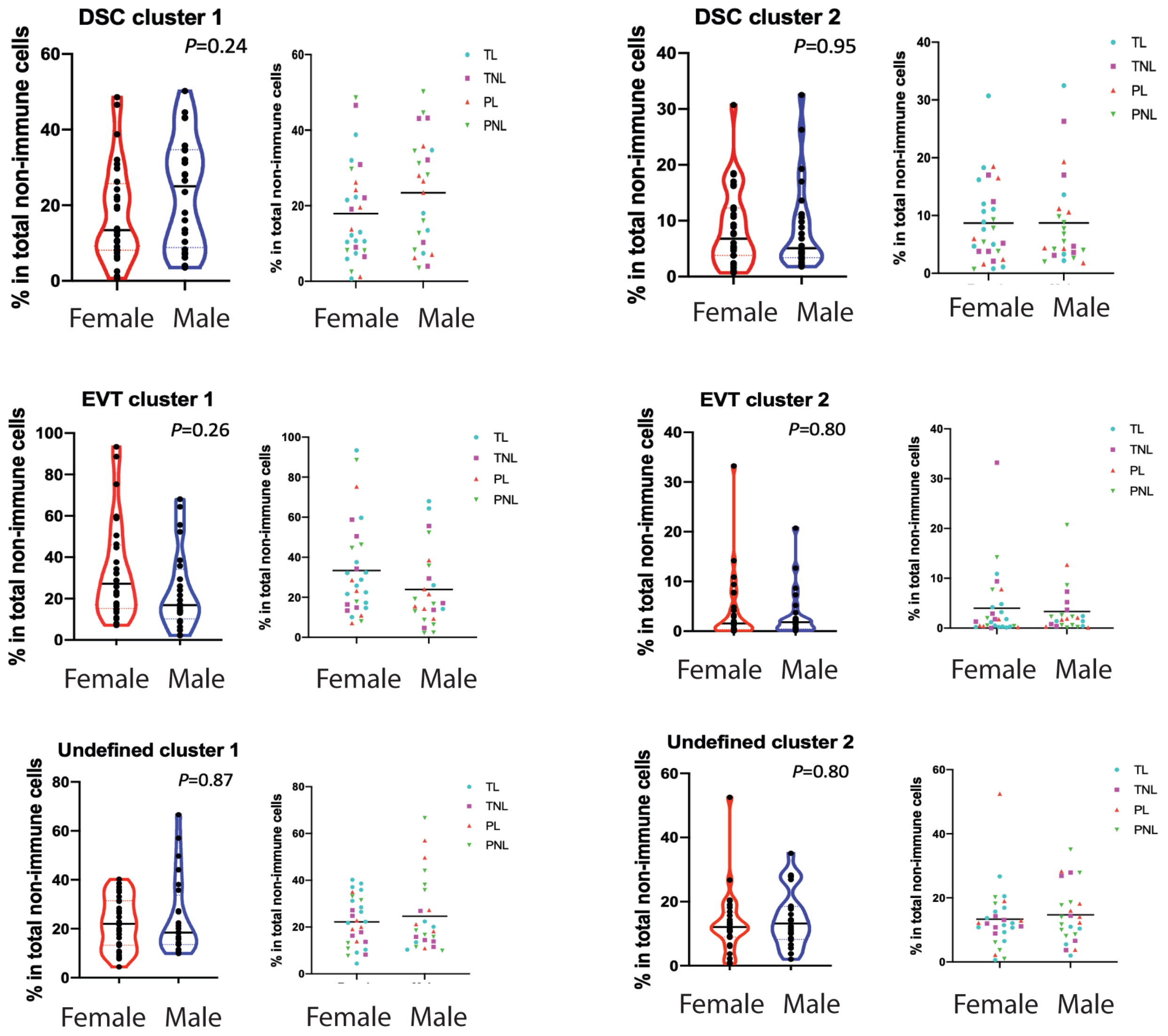

Figures S11. Correlations between gestational age and non-immune cell clusters (A) and by the four comparison groups (B).

A)

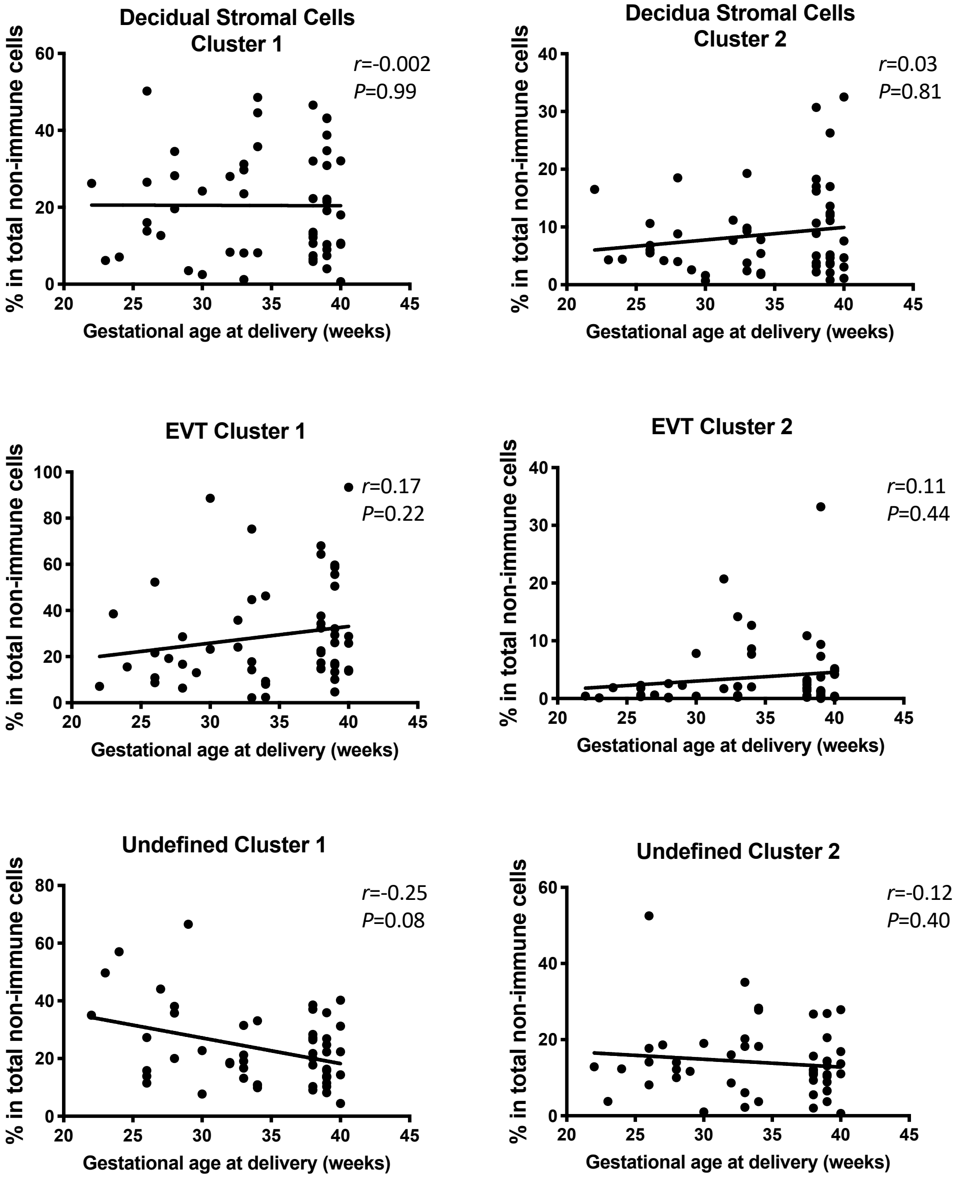

B)

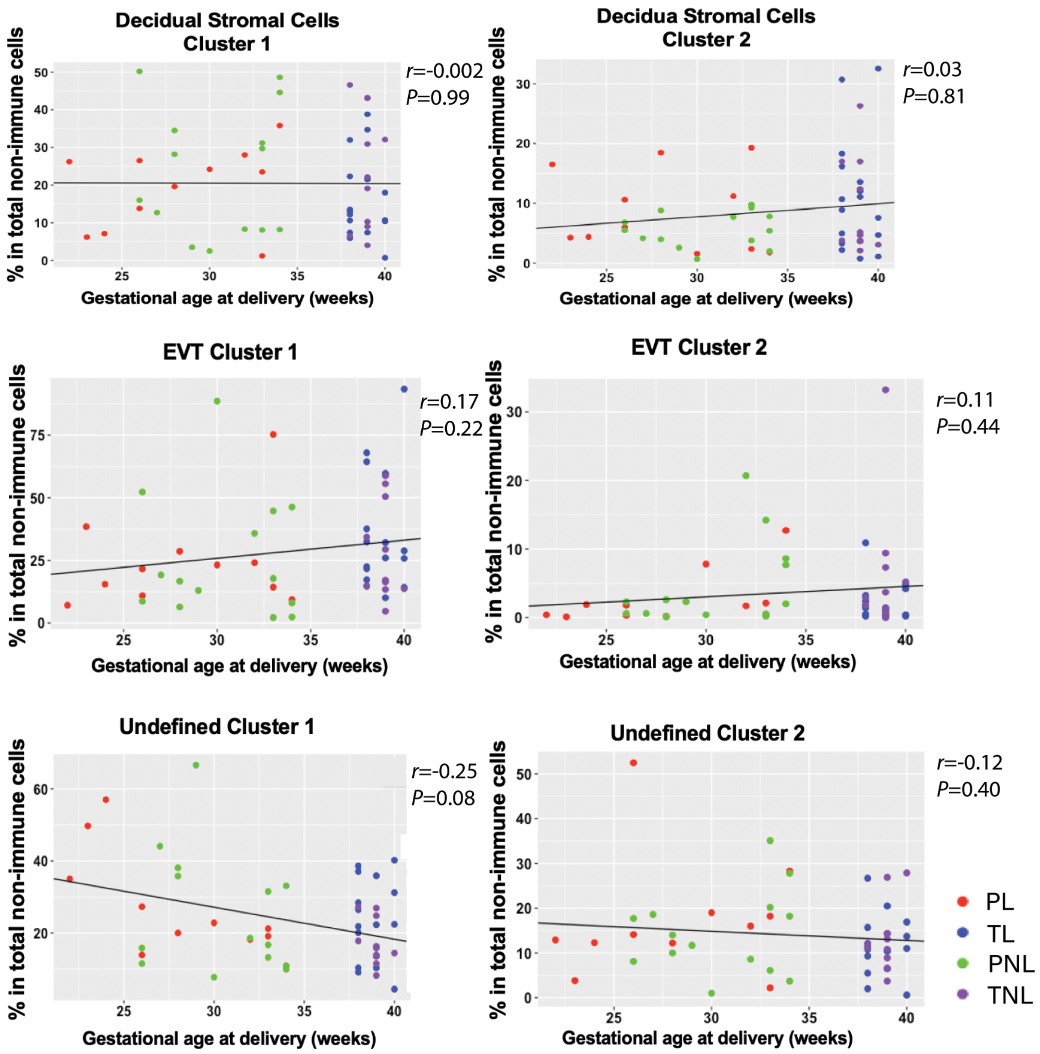
Decidual Stromal Cells
Cluster 1
r=-0.002
P=0.99
Decidua Stromal Cells
Cluster 2
r=0.03
P=0.81
EVT Cluster 1
r=0.17
P=0.22
EVT Cluster 2
r=0.11
P=0.44
Undefined Cluster 1
r=-0.25
P=0.08
Undefined Cluster 2
r=-0.12
P=0.40
% in total non-immune cells
Gestational age at delivery (weeks)
PL
TL
PNL
TNL

Figures S12. PD-L1+ non-immune cells by infant sex (A) and by the four comparison groups (B). *P*-value from Robust Rank-Order test.

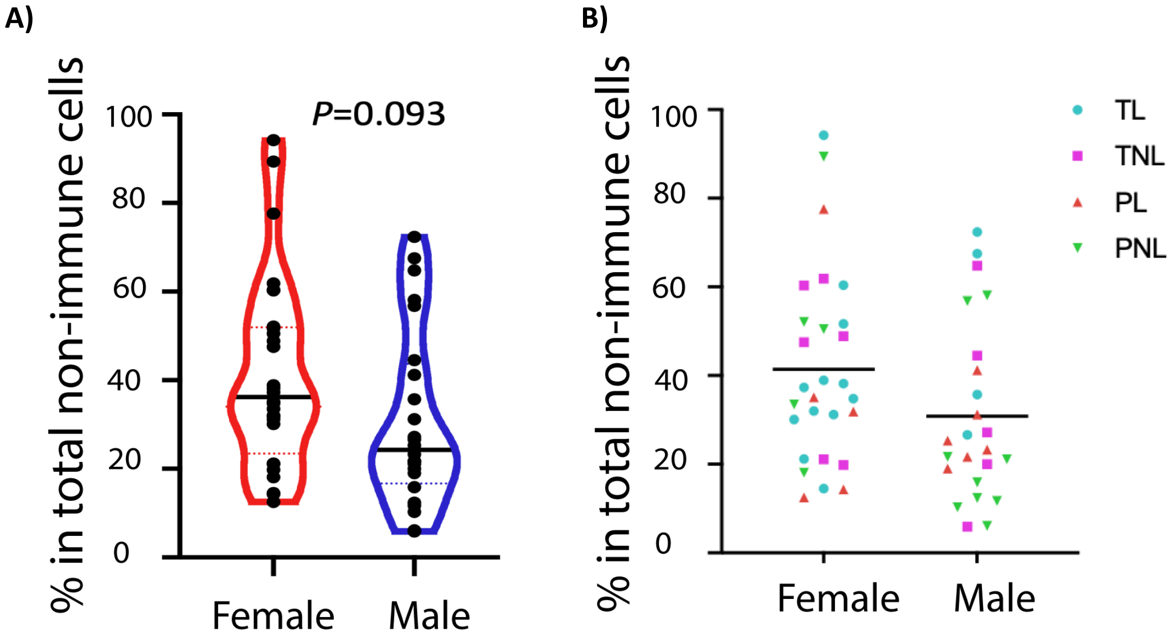

Figures S13. PD-L1^{+} non-immune cells by gestational age and infant sex and in laboring and non-laboring pregnancies.

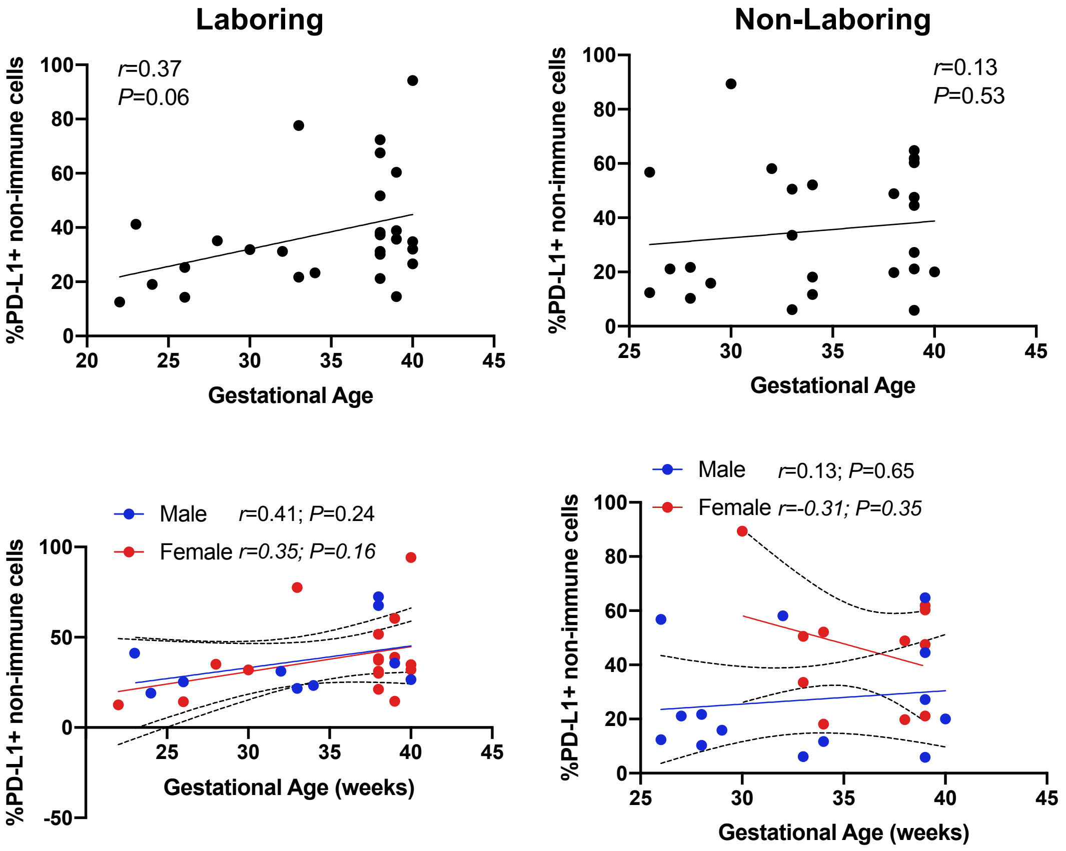

References

1. T. Hollt *et al.*, Cytosplore: Interactive Immune Cell Phenotyping for Large Single-Cell Datasets. *Comput Graph Forum* **35**, 171-180 (2016).

2. V. van Unen *et al.*, Visual analysis of mass cytometry data by hierarchical stochastic neighbour embedding reveals rare cell types. *Nat Commun* **8**, 1740 (2017).

3. H. Chen *et al.*, Cytofkit: A Bioconductor Package for an Integrated Mass Cytometry Data Analysis Pipeline. *PLoS Comput Biol* **12**, e1005112 (2016).

4. E. A. Enninga, W. K. Nevala, D. J. Creedon, S. N. Markovic, S. G. Holtan, Fetal sex-based differences in maternal hormones, angiogenic factors, and immune mediators during pregnancy and the postpartum period. *Am J Reprod Immunol* **73**, 251-262 (2015).

5. A. Ghidini, C. M. Salafia, Gender differences of placental dysfunction in severe prematurity. *BJOG* **112**, 140-144 (2005).

6. S. Cvitic *et al.*, The human placental sexome differs between trophoblast epithelium and villous vessel endothelium. *PLoS One* **8**, e79233 (2013).

7. S. Kim-Fine *et al.*, Male gender promotes an increased inflammatory response to lipopolysaccharide in umbilical vein blood. *J Matern Fetal Neonatal Med* **25**, 2470-2474 (2012).
